# High-Pressure Rate Rules for Ether Alkylperoxy Radical Isomerization

**DOI:** 10.1021/acs.jpca.4c04840

**Published:** 2024-09-17

**Authors:** Samah Y. Mohamed, Yeonjoon Kim, Gina Fioroni, Seonah Kim, Robert McCormick

**Affiliations:** † National Renewable Energy Laboratory, Golden, Colorado 80401, United States; ‡ Chemistry Department, Colorado State University, Fort Collins, Colorado 80523, United States

## Abstract

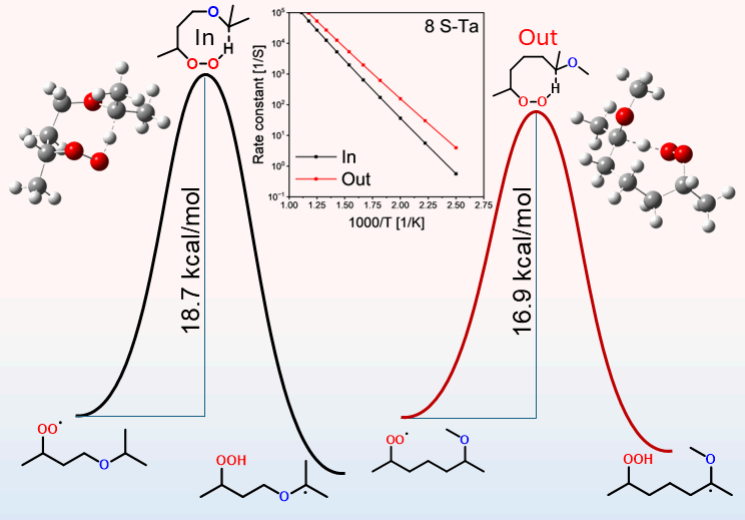

The first isomerization reaction of an alkylperoxy (RO_2_) radical holds significant importance in low-temperature oxidation, as it governs the branching ratios of the hydroperoxyalkyl (QOOH) radicals, which influence the competition between the chain-propagation and chain-branching reactions. In this study, we systematically calculated high-pressure rate rules for the RO_2_ isomerization reaction of monoethers, exploring 5-, 6-, 7-, and 8-membered ring transition states. Primary, secondary, and tertiary carbon sites, where both the abstracting peroxy group and the abstracted hydrogen are located, were considered, with particular emphasis on distinguishing between secondary carbons adjacent (alpha) and nonadjacent to the ether functional group. Using the G4//B3LYP/6-311++G(2df,2pd) level of theory and the transition state theory, we estimated the rate constants and the Arrhenius coefficient for over 120 possible isomerization reactions. We examined the effect of ring size and ring atoms, revealing that 6- and 7-membered ring isomerizations were generally the fastest. The impact of the ether functional group on transition states was investigated by comparing reactions with identical ring size, peroxy, and radical positions, but with the ether functional group positioned either outside (i.e., *out*) or inside (i.e., *in*) the transition state ring, leading to differences in the rate constants. When comparing to analogous alkane rate constants, differences of up to an order of magnitude were observed, underscoring the need for caution when assigning rate rules by analogy. We applied our rate constants in the di-iso-butyl ether kinetic model and evaluated their influence on low-temperature chemistry finding that they altered the branching ratios by up to a factor of 9, highlighting the significance of site-specific rate constants for more accurate low-temperature modeling.

## 1 Introduction

Diesel-boiling-range ethers (>C8) are promising diesel blend stocks due to their compatibility with diesel fuel, high cetane number -in many cases more than 100- and lower particulate emissions.^1−5^ They can also be produced from biomass with potentially more than 50% life cycle greenhouse gas emissions reductions relative to conventional fuels.^6,7^ Investigating their combustion characteristics and behavior under engine conditions requires a thorough understanding of their kinetics and chemistry. Kinetic mechanisms for higher-molecular-weight ethers have been developed based on analogies to alkane or alcohol chemistry,^1,8^ with modified activation energies,^2^ but this approach may introduce inaccuracy, particularly for low-temperature reactions at the alpha and beta carbons to the ether functional group. Because of these compounds’ very high cetane numbers, they are very reactive at low temperatures, and kinetic mechanisms are highly sensitive to these reactions.

Calculated rate rules for higher-molecular-weight ethers have been limited to high-temperature reactions of H-abstractions and beta-scission.^8−11^ However, the kinetics of alkylperoxy (RO_2_) and hydroperoxyalkylperoxy (OOQOOH) radicals play a critical role in the fuel low-temperature combustion chemistry.^12^ Two important steps for chain-branching and low-temperature chemistry are the first isomerization RO_2_ → QOOH and the second isomerization ȮOQOOH → KHP + OH reactions.^13^ The first isomerization reaction of the RO_2_ radical is especially significant because it determines the branching ratios of the QOOH radicals, which can favor either chain-propagation reactions (decreasing reactivity) or chain-branching reactions (increasing reactivity). Nonetheless, the first isomerization rate rules have only been calculated for short-chain diethyl ether (DEE), which limits the availability of site-specific rate rules for longer chains.

In a mechanistic exploration of DEE combustion, Di Tommaso et al.^14^ found that RO_2_ isomerization is a major pathway and plays an important role in DEE oxidation. Subsequently, a series of computational studies were performed for this reaction. Sakai et al.^15^ calculated the isomerization rate constants at CBS-QB3//B3LYP/CBSB7 using transition state theory and Eckart tunneling. They found that the structural environment around the ether oxygen atom affected the rate constants and made the isomerization 3–8 times faster than for similarly located reactive sites in an alkane at 700 K. More recently, Danilack et al.^16^ performed higher-level master equation calculations at CCSD(T)-F12b/cc-pVTZ-F12//B2PLYPD3/cc-pVTZ to calculate the thermal low-temperature oxidation of DEE and investigate the stereometric effects in the oxidation mechanism. Further studies examined the impact of incorporating nonthermal (non-Boltzmann) reactions in DEE low-temperature chemistry.^17,18^ They demonstrated that including these pathways decreased the ignition delay time of DEE by a factor of 2.4^17^ and increased the mechanism reactivity compared to flow reactor and jet-stirred reactor data.^18^ Xing et al.^12^ calculated the first isomerization rate constants using the multistructural canonical variational transition state theory with CCSD(T)-F12a/jun-cc-pVDZ//M05-2X/jun-cc-pVTZ. This study showed slower rates than Sakai et al.,^15^ which is attributed to the multistructural effect. Duan et al.^19^ revisited the rate constants for the isomerization of DEE RO_2_ radicals using multistructural torsional variational transition state theory with small curvature tunneling at the CCSD(T)/aug-cc-pVTZ//M06-2X/cc-pVTZ level of theory. They stated that their approach led to more accurate rate constants, resulting in a relatively better agreement for DEE ignition delay time but barely affecting jet-stirred reactor speciation. However, the Sakai et al.^15^ rate constants showed better agreement for low-pressure ignition delay time.

Studies have shown that the ether functional group affects the calculated rate constants, and therefore, analogous rate constants from alkane or alcohol mechanisms should be applied with caution.^19^ Given the limited availability of calculated site-specific rate constants in the literature, the increasing interest in higher ethers as diesel blend stocks, and the importance of understanding their autoignition characteristics, we conducted systematic calculations for the first isomerization of RO_2_ radicals in low-temperature autoignition chemistry.

This study determined the rate parameters for RO_2_ isomerization to QOOH via 5-, 6-, 7-, and 8-membered rings, with all possible peroxy and radical positions for primary, secondary, and tertiary carbons. Alpha peroxy or radicals to the ether functional group were also distinguished.

## 2 Methods

**Figure 1:**
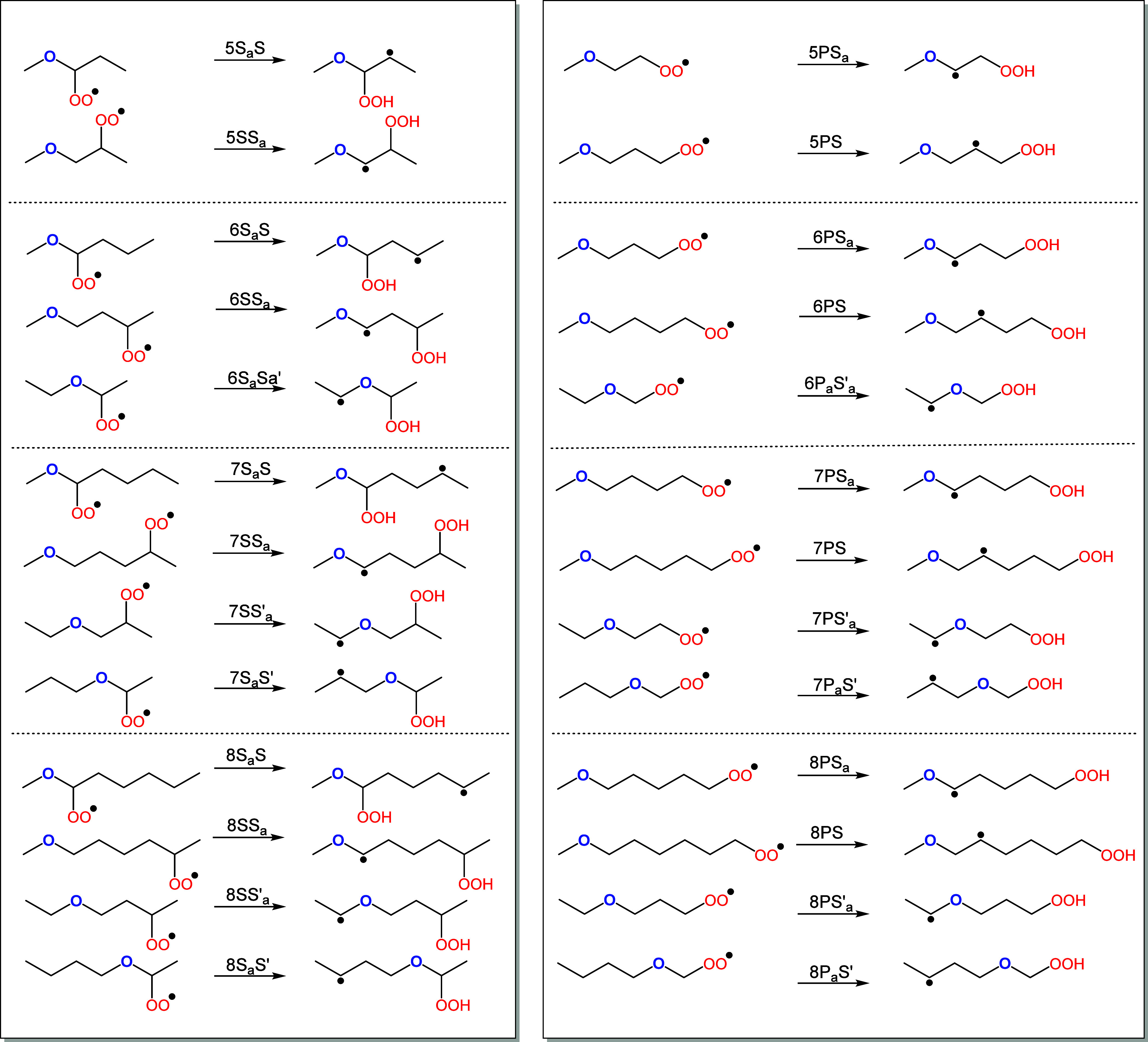
Example of the reactions’ nomenclature for the NS-S and NP-S reactions (*N* = 5, 6, 7, 8). For example, 5PS_a_ is a five-membered ring isomerization where the peroxy group is located on a primary carbon and the abstracted hydrogen is from a secondary carbon that is alpha to the ether functional group.

In this study, we considered the isomerization of different alkylperoxy radicals (RO_2_) to account for all possible ether site-specific reactions. Isomerizations are denoted as NX-*Y*, where *N* can be 5, 6, 7, or 8, representing the transition ring size (i.e., the number of atoms in the transition state ring). *X* and *Y* represent the chemical nature of the carbons where the peroxy and abstracted radical sites are located, respectively. *X* and *Y* can be primary (P), secondary (S), or tertiary (T). The *a* subscript will be added for *X* and/or *Y* whenever applicable to denote an alpha site to the ether functional group. The peroxy and abstracted radical sites can be situated on the same side of the ether functional group or on opposite sides separated by the ether group. In the latter case, *Y*′ will be used. Examples of this nomenclature are illustrated in Figure 1 for all considered membered rings with secondary peroxy and secondary radical sites (S–S) and primary peroxy and secondary radical sites (P–S). A complete list of the calculated RO_2_ reactants is illustrated in the Supporting Information.

The automated generation of possible isomerization reactions was carried out using the RDKit cheminformatics package^20^ implemented in Python 3.8.^21^ We generated the simplified molecular-input line-entry system (SMILES) strings of ether reactants and SMILES arbitrary target specification (SMARTS) strings of isomerization reactions. Next, SMILES strings of possible RO_2_ radicals were created from the reactants’ SMILES strings and used as inputs for the SMARTS pattern-matching algorithm. If the given RO_2_ radical has matching substructures with the reaction SMARTS, the SMILES strings of corresponding QOOH products are extracted. Such generation methodology was inspired by the combinatorial enumeration method of possible reaction intermediates based on molecular graphs.^22^ Source code and raw data are available via a GitHub repository.^23^

Gaussian 16^24^ was employed for all calculations of reactants, products, and transition states. Conformational analysis was performed for transition states by rotating all C–O and C–C hindered rotors by 120°, generating 3^*n*^ conformers, where *n* is the number of rotors. Automated Quantum Mechanical Environments (AQME)^25^ software was utilized to convert SMILES strings into 3D geometries by generating all possible conformers of reactants and products, and to automatically create Gaussian 16 input files. To locate the minimum energy conformer for all reactants, products, and transition states, generated conformers were optimized using the B3LYP/6-31+G(d,p) method. Conformers within 0.5 kcal/mol of the minimum energy conformer were optimized again at B3LYP/6-311++G(2df,2pd), and frequency calculations were performed at the same level of theory, applying a scaling factor of 0.9679.^26^ The energies of the lowest-energy conformers were calculated at the G4^27^ level of theory, which has a root mean squared deviation (an approximate 95% confidence interval) of 0.94 kcal/mol compared to values from the Active Thermochemical Tables (ATcT).^28^ Given the large number of species and transition states considered in this study, the less computationally demanding B3LYP functional was chosen for geometry optimization. While more accurate functionals are available, B3LYP demonstrated reasonable performance in predicting minimum energy conformers, bond lengths and angles.^29^ Likewise, to accommodate the extensive number of targeted species, atomization was used to estimate the enthalpy of formation at 298 K. The estimated enthalpies are given in the Supporting Information. The isodesmic reactions, known for their higher accuracy,^30^ were applied to selected species to compare their energy values with those obtained by atomization. The observed differences in energy values ranged from 0.5 to 1.2 kcal/mol, as detailed in the Supporting Information. Hindered rotor (HR) treatment was included for all reactants, products, and transition states of the 5- and 6-membered ring isomerization. Relaxed scans in 10° increments of all hindered rotors were calculated at the B3LYP/6-31+G(d,p) level of theory to calculate the rotational barriers using the Pitzer–Gwinn method.^31^ HR barriers were assigned for the 7- and 8-membered ring isomerization species and transition states in analogy to the 5- and 6-membered rings due to the large number of rotors in these systems. We calculated the rotational barriers for the smaller rings (5- and 6-membered ring), categorized the rotors into specific groups, and averaged the values within each group. We then assigned these averaged rotational barriers to the corresponding rotors in the larger rings (7- and 8-membered ring). Transition states were confirmed by the single imaginary frequency, and the intrinsic reaction coordinate (IRC) calculations performed at the B3LYP/6-31+G(d,p) level of theory. Eckart tunneling was assumed and computed from the barrier width from the IRC calculations. Classical transition state theory (CTST) was used for rate constants calculations as implemented in ChemRate,^32^ represented by the Eyring equation , where κ(*T*) is the tunneling transmission coefficient, *k*_B_ is Boltzmann’s constant, *h* is Planck’s constant, *T* is the temperature, *R* is the ideal gas constant, Δ*S*^‡^ and Δ*H*^‡^ are, respectively, the entropy and enthalpy difference between the transition state and the reactant. The equation was fitted over the temperature range 300–1500 K to obtain the Arrhenius parameters (*A*, *n*, and *E*_a_) in the Arrhenius equation, , where *A* is the pre-exponential factor, *n* is the temperature fitting parameter, *T* is temperature, and *E*_a_ is the activation energy.

## 3 Results

The minimum energy conformers of all reactants, products, and transition states were determined and used to calculate rate constants. Different stereoisomers were considered in cases where two chiral centers existed, and only the most stable diastereomers were used. High-pressure-limit rate constants were fitted to the modified Arrhenius equation in the temperature range 300–1500 K, as detailed in Table 1, on a hydrogen basis. Table 1 also shows the reaction barriers or barrier height that is calculated as the difference between the transition state and the reactant enthalpy of formation. The more strained 5-membered ring transition state has relatively the highest barrier. A previous study reported that 6- and 7-membered ring barriers have almost the same magnitude, which was attributed to the latter having a pseudo 6-membered ring where the transferring hydrogen between the radical site and the abstracting oxygen (O···H–C) formed an angle of 180°.^33^ Interestingly, we found the energy barriers for 6-, 7-, and 8-membered rings to be of the same magnitude, with the latter exhibiting even lower barriers, especially when the abstracted radical is in a tertiary site. One possible reason for this barrier height trend is the error associated with the atomization method used for enthalpy of formation calculations. The error in atomization is known to be proportional to the size of the species considered,^30^ and therefore, more significant errors are expected for the 8-membered ring isomerization.

**Table 1:** Reaction Barriers (kcal/mol) and High-Pressure-Limit Rate Parameters for RO2 Isomerization Reaction Fit between 300 and 1500 K, on a Hydrogen Basis^a^

**reaction**	**(OO)-rad**^b^	***A***	***n***	***E*_a_**	**reaction barrier**^c^
5-Membered ring (*N* = 5)					
[O]OCCOC → CO[CH]COO	P–S_a_	4.37 × 10^–02^	3.80	22.39	29.70
[O]OCC(C)OC → CO[C](C)COO	P–T_a_	8.91 × 10^02^	2.54	21.77	26.27
[O]OC(OC)C → [CH2]C(OC)OO	S_a_–P	7.41 × 10^–04^	4.13	28.49	36.03
[O]OC(CC)OC → C[CH]C(OC)OO	S_a_–S	1.82 × 10^–01^	3.63	25.81	32.65
[O]OC(C)COC → CO[CH]C(C)OO	S–S_a_	1.74 × 10^00^	3.25	22.08	28.20
[O]OC(C)C(C)OC → CO[C](C)C(C)OO	S–T_a_	1.45 × 10^03^	2.46	21.11	26.03
[O]OC(OC)C(C)C → COC(OO)[C](C)C	S_a_–T	1.41 × 10^02^	2.79	24.03	29.63
[O]OC(C)(OC)C → [CH2]C(C)(OC)OO	T_a_–P	5.13 × 10^–11^	6.23	21.52	34.57
[O]OC(C)(C)COC → CO[CH]C(C)(C)OO	T–S_a_	4.17 × 10^08^	0.84	28.30	28.43
[O]OC(OC)(C)CC → C[CH]C(C)(OC)OO	T_a_–S	2.00 × 10^–02^	3.73	23.75	30.94
[O]OC(C)(OC)C(C)C → COC(C)(OO)[C](C)C	T_a_–T	2.34 × 10^00^	3.22	22.08	27.94
[O]OC(C)(C)C(C)OC → CO[C](C)C(C)(C)OO	T–T_a_	2.63 × 10^00^	3.17	20.98	26.83
6-Membered ring (*N* = 6)					
COCO[O] → [CH2]OCOO	P_a_–P_a_′	1.95 × 10^02^	2.45	17.7	21.32
CCOCO[O] → C[CH]OCOO	P_a_–S_a_′	7.76 × 10^03^	2.06	15.92	19.28
COCCCO[O] → CO[CH]CCOO	P–S_a_	2.82 × 10^02^	2.59	15.80	20.57
CC(C)OCO[O] → C[C](C)OCOO	P_a_–T_a_′	2.82 × 10^04^	1.91	14.40	17.56
COC(C)CCO[O] → CO[C](C)CCOO	P–T_a_	9.77 × 10^01^	2.47	15.54	19.94
CCC(OC)O[O] → [CH2]CC(OC)OO	S_a_–P	1.23 × 10^01^	2.69	19.79	24.47
COC(C)O[O] → [CH2]OC(C)OO	S_a_–P_a_′	1.12 × 10^02^	2.34	17.31	20.97
COCCC(C)O[O] → CO[CH]CC(C)OO	S–S_a_	2.69 × 10^01^	2.64	14.90	20.09
COC(O[O])CCC → C[CH]CC(OC)OO	S_a_–S	2.24 × 10^01^	2.71	17.33	22.16
CC(O[O])OCC → C[CH]OC(C)OO	S_a_–S_a_′	2.57 × 10^04^	1.84	16.00	18.97
COC(C)CC(C)O[O] → CO[C](C)CC(C)OO	S–T_a_	5.75 × 10^02^	2.21	15.28	19.51
COC(O[O])CC(C)C → COC(C[C](C)C)OO	S_a_–T	1.38 × 10^03^	2.00	16.32	19.91
CC(C)OC(C)O[O] → C[C](C)OC(C)OO	S_a_–T_a_′	4.57 × 10^05^	1.50	14.67	17.33
COC(C)(O[O])CC → COC(C)(OO)C[CH2]	T_a_–P	7.76 × 10^–02^	3.12	17.06	22.97
COC(C)(C)O[O] → [CH2]OC(C)(C)OO	T_a_–P_a_′	1.38 × 10^01^	2.64	16.37	20.81
COCCC(C)(C)O[O] → CO[CH]CC(C)(C)OO	T–S_a_	1.55 × 10^01^	2.59	14.12	19.83
COC(C)(O[O])CCC → C[CH]CC(C)(OC)OO	T_a_–S	1.48 × 10^00^	2.73	15.19	20.70
CCOC(C)(C)O[O] → C[CH]OC(C)(C)OO	T_a_–S_a_′	4.57 × 10^02^	2.27	14.46	18.92
COC(C)CC(C)(O[O])C → CO[C](C)CC(C)(C)OO	T–T_a_	2.69 × 10^01^	2.44	14.53	19.40
COC(C)(O[O])CC(C)C → COC(C)(C[C](C)C)OO	T_a_–T	4.79 × 10^03^	1.64	14.15	17.68
CC(C)OC(C)(C)O[O] → C[C](C)OC(C)(C)OO	T_a_–T_a_′	1.07 × 10^03^	2.07	14.24	18.43
7-Membered ring (*N* = 7)					
[O]OCCOC → [CH2]OCCOO	P–P_a_′	1.20 × 10^01^	2.62	16.75	21.89
[O]OCOCC → [CH2]COCOO	P_a_–P′	1.12 × 10^–01^	3.06	18.38	23.79
[O]OCOCCC → C[CH]COCOO	P_a_–S′	2.00 × 10^01^	2.71	16.01	20.83
[O]OCCOCC → C[CH]OCCOO	P–S_a_′	1.86 × 10^02^	2.22	15.48	19.68
[O]OCCCCOC → CO[CH]CCCOO	P–S_a_	1.41 × 10^00^	3.04	12.84	18.92
[O]OCCOC(C)C → C[C](C)OCCOO	P–T_a_′	1.66 × 10^02^	2.25	15.02	18.57
[O]OCCCC(C)OC → CO[C](C)CCCOO	P–T_a_	2.00 × 10^02^	2.35	12.64	17.15
[O]OCOCC(C)C → C[C](C)COCOO	P_a_–T′	4.57 × 10^02^	2.06	14.35	17.85
[O]OC(C)OCC → [CH2]COC(C)OO	S_a_–P′	1.55 × 10^00^	2.72	19.55	24.17
[O]OC(OC)CCC → [CH2]CCC(OC)OO	S_a_–P	9.55 × 10^–02^	3.07	18.65	24.15
[O]OC(C)COC → [CH2]OCC(C)OO	S–P_a_′	1.26 × 10^–01^	2.96	16.24	21.83
[O]OC(C)COCC → C[CH]OCC(C)OO	S–S_a_′	8.51 × 10^01^	2.13	15.38	19.71
[O]OC(C)CCCOC → CO[CH]CCC(C)OO	S–S_a_	7.24 × 10^–02^	3.15	12.70	19.19
[O]OC(C)OCCC → C[CH]COC(C)OO	S_a_–S′	2.95 × 10^01^	2.50	16.83	21.30
[O]OC(OC)CCCC → C[CH]CCC(OC)OO	S_a_–S	1.70 × 10^00^	2.84	16.11	21.29
[O]OC(C)COC(C)C → C[C](C)OCC(C)OO	S–T_a_′	7.94 × 10^02^	1.98	15.09	18.60
[O]OC(C)CCC(C)OC → CO[C](C)CCC(C)OO	S–T_a_	2.88 × 10^01^	2.40	12.62	17.38
[O]OC(C)OCC(C)C → C[C](C)COC(C)OO	S_a_–T′	1.07 × 10^03^	1.87	15.13	18.30
[O]OC(OC)CCC(C)C → COC(CC[C](C)C)OO	S_a_–T	3.31 × 10^02^	2.21	14.60	18.47
[O]OC(C)(C)COC → [CH2]OCC(C)(C)OO	T–P_a_′	5.13 × 10^–02^	3.06	16.66	22.70
[O]OC(C)(C)OCC → [CH2]COC(C)(C)OO	T_a_–P′	2.34 × 10^–02^	3.18	18.83	25.13
[O]OC(OC)(C)CCC → [CH2]CCC(C)(OC)OO	T_a_–P	2.69 × 10^–06^	4.15	14.10	23.20
[O]OC(C)(C)COCC → C[CH]OCC(C)(C)OO	T–S_a_′	3.02 × 10^02^	2.14	15.93	20.50
[O]OC(C)(C)CCCOC → CO[CH]CCC(C)(C)OO	T–S_a_	7.94 × 10^–04^	3.70	12.17	20.01
[O]OC(C)(C)OCCC → C[CH]COC(C)(C)OO	T_a_–S′	5.25 × 10^–01^	2.87	16.77	22.70
[O]OC(OC)(C)CCCC → C[CH]CCC(C)(OC)OO	T_a_–S	2.34 × 10^–01^	2.96	14.40	20.30
[O]OC(C)(C)COC(C)C → C[C](C)OCC(C)(C)OO	T–T_a_′	2.04 × 10^03^	1.89	15.66	19.34
[O]OC(C)(C)CCC(C)OC → CO[C](C)CCC(C)(C)OO	T–T_a_	1.20 × 10^00^	2.82	12.46	18.23
[O]OC(C)(C)OCC(C)C → C[C](C)COC(C)(C)OO	T_a_–T′	3.16 × 10^02^	2.10	15.36	19.67
[O]OC(OC)(C)CCC(C)C → COC(C)(CC[C](C)C)OO	T_a_–T	4.17 × 10^01^	2.33	13.16	17.72
8-Membered ring(*N* = 8)					
[O]OCOCCC → [CH2]CCOCOO	P_a_–P′	2.63 × 10^00^	2.80	17.47	22.06
[O]OCCCOC → [CH2]OCCCOO	P–P_a_′	1.15 × 10^–01^	3.15	15.68	21.61
[O]OCCCOCC → C[CH]OCCCOO	P–S_a_′	2.04 × 10^01^	2.33	14.95	19.67
[O]OCCCCCOC → CO[CH]CCCCOO	P–S_a_	3.72 × 10^–05^	3.29	11.76	17.76
[O]OCOCCCC → C[CH]CCOCOO	P_a_–S′	3.89 × 10^01^	2.35	15.28	19.23
[O]OCCCOC(C)C → C[C](C)OCCCOO	P–T_a_′	4.90 × 10^–01^	2.32	14.38	18.57
[O]OCCCCC(C)OC → CO[C](C)CCCCOO	P–T_a_	4.27 × 10^–01^	2.21	12.47	16.64
[O]OCOCCC(C)C → C[C](C)CCOCOO	P_a_–T′	5.25 × 10^01^	1.89	13.53	16.50
[O]OC(C)OCCC → [CH2]CCOC(C)OO	S_a_–P′	7.94 × 10^00^	2.58	18.53	22.73
[O]OC(CCCC)OC → [CH2]CCCC(OC)OO	S_a_–P	5.50 × 10^–02^	3.09	17.81	23.47
[O]OC(C)CCOC → [CH2]OCCC(C)OO	S–P_a_′	1.86 × 10^–02^	3.30	15.34	21.72
[O]OC(C)CCOCC → C[CH]OCCC(C)OO	S–S_a_′	5.37 × 10^01^	2.11	15.44	19.80
[O]OC(C)CCCCOC → CO[CH]CCCC(C)OO	S–S_a_	1.15 × 10^00^	2.65	13.85	19.07
[O]OC(C)OCCCC → C[CH]CCOC(C)OO	S_a_–S′	2.63 × 10^02^	2.18	16.33	19.90
[O]OC(CCCCC)OC → C[CH]CCCC(OC)OO	S_a_–S	1.41 × 10^00^	2.77	15.55	20.61
[O]OC(C)CCOC(C)C → C[C](C)OCCC(C)OO	S–T_a_′	6.31 × 10^00^	2.59	14.27	18.69
[O]OC(C)CCCC(C)OC → CO[C](C)CCCC(C)OO	S–T_a_	5.01 × 10^01^	2.23	12.65	16.94
[O]OC(C)OCCC(C)C → C[C](C)CCOC(C)OO	S_a_–T′	3.39 × 10^03^	1.84	14.20	17.18
[O]OC(CCCC(C)C)OC → COC(CCC[C](C)C)OO	S_a_–T	4.90 × 10^01^	2.11	14.05	17.71
[O]OC(C)(C)OCCC → [CH2]CCOC(C)(C)OO	T_a_–P′	1.95 × 10^00^	2.58	17.00	21.74
[O]OC(C)(CCCC)OC → [CH2]CCCC(C)(OC)OO	T_a_–P	4.37 × 10^–03^	3.30	16.29	22.76
[O]OC(C)(C)CCOC → [CH2]OCCC(C)(C)OO	T–P_a_′	5.25 × 10^–03^	3.31	13.94	20.38
[O]OC(C)(C)CCOCC → C[CH]OCCC(C)(C)OO	T–S_a_′	1.29 × 10^01^	2.50	13.27	18.46
[O]OC(C)(C)CCCCOC → CO[CH]CCCC(C)(C)OO	T–S_a_	9.55 × 10^–02^	2.97	11.61	17.68
[O]OC(C)(C)OCCCC → C[CH]CCOC(C)(C)OO	T_a_–S′	1.74 × 10^01^	2.30	14.45	18.95
[O]OC(C)(CCCCC)OC → C[CH]CCCC(C)(OC)OO	T_a_–S	3.39 × 10^–01^	2.87	14.21	19.91
[O]OC(C)(C)CCOC(C)C → C[C](C)OCCC(C)(C)OO	T–T_a_′	4.17 × 10^01^	2.29	12.72	17.15
[O]OC(C)(C)CCCC(C)OC → CO[C](C)CCCC(C)(C)OO	T–T_a_	1.35 × 10^06^	1.00	15.90	16.48
[O]OC(C)(C)OCCC(C)C → C[C](C)CCOC(C)(C)OO	T_a_–T′	6.17 × 10^03^	1.74	13.23	16.49
[O]OC(C)(CCCC(C)C)OC → COC(C)(CCC[C](C)C)OO	T_a_–T	1.35 × 10^02^	2.24	12.81	17.01
[O]OCCOCC → [CH2]COCCOO	P–P′	3.98 × 10^00^	2.50	20.81	25.33
[O]OCCOCCC → C[CH]COCCOO	P–S′	1.23 × 10^03^	2.07	18.28	22.56
[O]OCCOCC(C)C → C[C](C)COCCOO	P–T′	2.34 × 10^03^	1.81	15.89	19.40
[O]OC(C)COCC → [CH2]COCC(C)OO	S–P′	7.76 × 10^–01^	2.78	21.67	25.12
[O]OC(C)COCCC → C[CH]COCC(C)OO	S–S′	2.24 × 10^01^	2.32	17.94	22.38
[O]OC(C)COCC(C)C → C[C](C)COCC(C)OO	S–T′	1.29 × 10^02^	2.13	15.74	19.15
[O]OC(C)(C)COCC → [CH2]COCC(C)(C)OO	T–P′	4.68 × 10^01^	2.18	20.84	24.88
[O]OC(C)(C)COCCC → C[CH]COCC(C)(C)OO	T–S′	1.74 × 10^03^	1.95	18.24	22.13
[O]OC(C)(C)COCC(C)C → C[C](C)COCC(C)(C)OO	T–T′	1.51 × 10^05^	1.40	16.02	18.81

a A is the pre-exponential factor in 1/(s·K^*n*^), n is the temperature fitting parameter, and *E*_a_ is the activation energy in kcal/mol.

b (OO)-Rad denotes the peroxy and abstracted radical sites (P, S, or T). The *a* subscript indicates an alpha site to the ether functional group, and ′ indicates that the peroxy and abstracted radical sites are situated on opposite sides, separated by the ether group.

c Energy barriers are the difference between the transition state and the reactants’ enthalpy of formation.

### 3.1 Effect of Transition State Ring Size and Ring Atoms on Rate Constants

**Figure 2:**
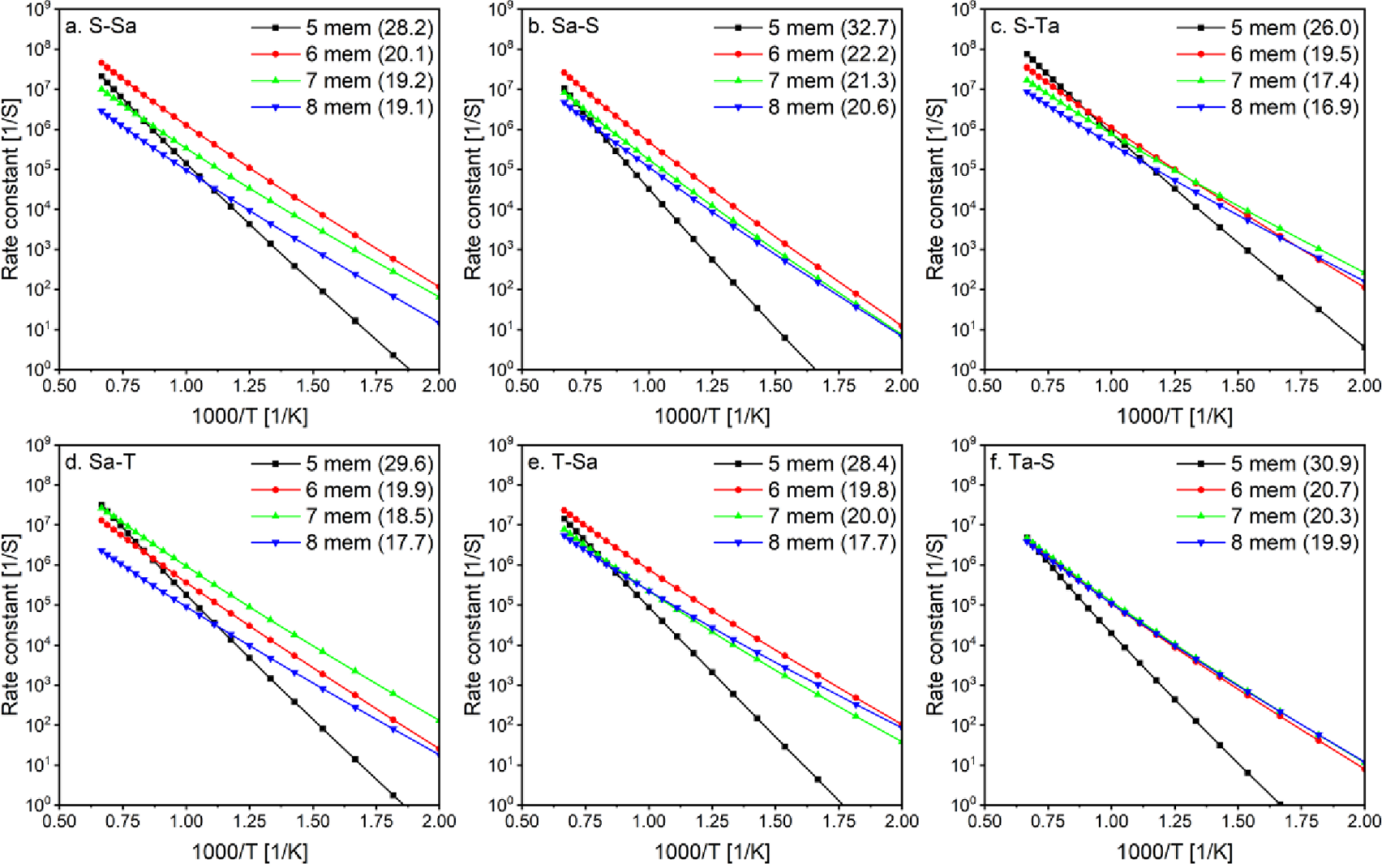
Effect of transition states ring size on calculated rate constants. Numbers between brackets are the barrier height for these reactions in kcal/mol. Isomerization nomenclature is explained in the Methods section.

The effect of the ring size is explored by examining the temperature dependence of selected rate constants, as shown in Figure 2. A general trend is observed where the highly strained 5-membered ring isomerizations have the lowest rate constants, consistent with the highest calculated barrier heights (Table 1). Reactions proceeding via 6-, 7-, and 8-membered rings exhibited similar magnitudes, especially at lower temperatures due to their close energy barrier values. However, the 8-membered ring is slower at higher temperatures as it locks up more rotors, reducing entropy and lowering the rate constants. The 6-membered ring is typically the fastest, though in some cases, the 7-membered ring can be faster than the 6-membered ring, especially at lower temperatures.

**Figure 3:**
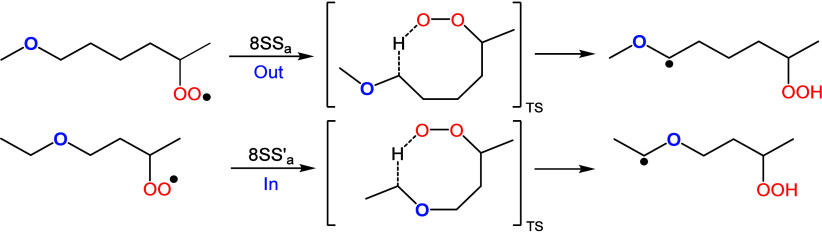
Scheme of two 8S–S_a_ reactions proceeding by abstracting hydrogen from different sides of the ether functional group (*Out* or *In*).

**Figure 4:**
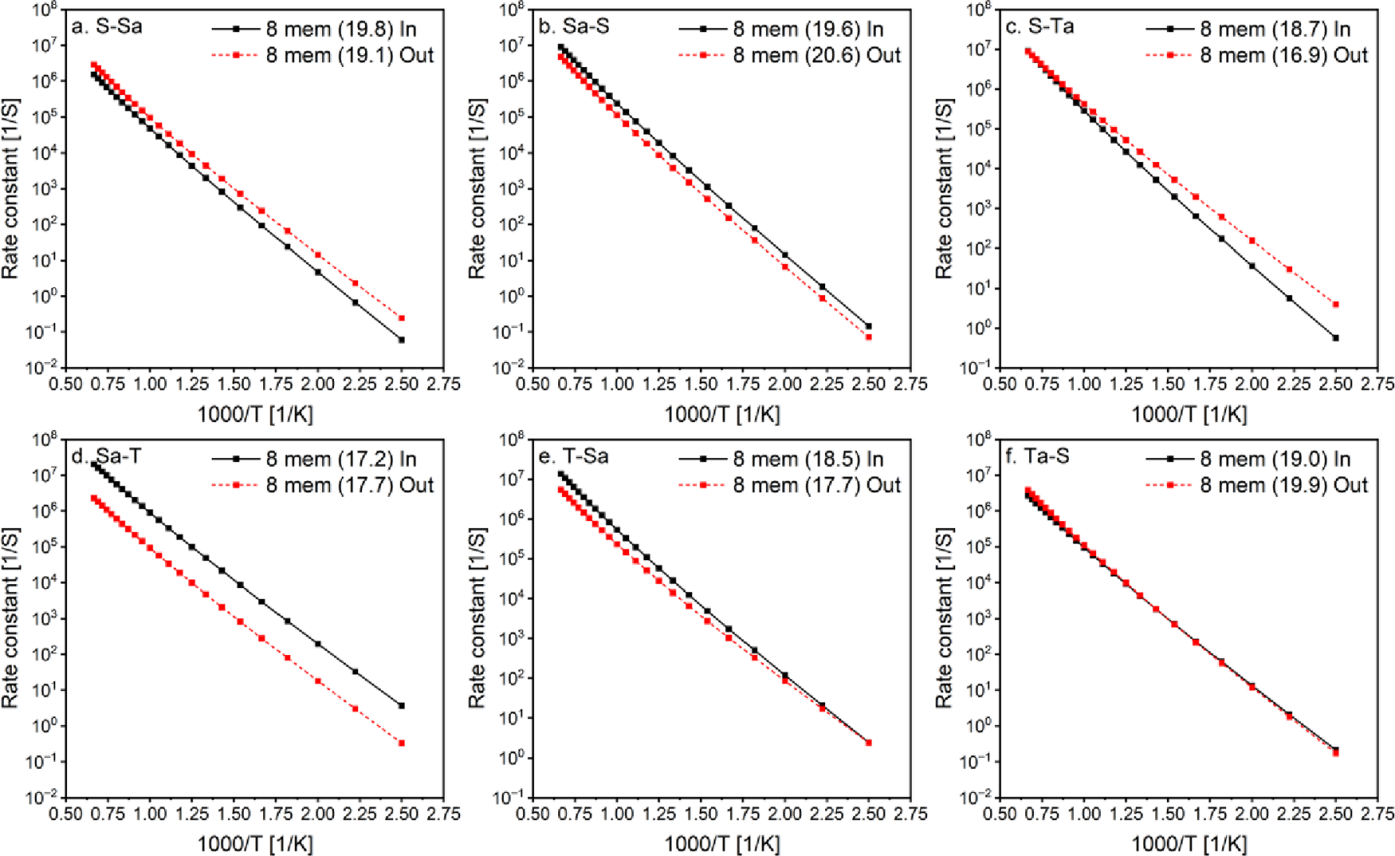
Effect of transition state structure on calculated rate constants. *In* or *Out* indicates the ether functional group inside or outside the transition state ring, respectively. Numbers between brackets are the barrier height for these reactions in kcal/mol. Isomerization nomenclature is explained in the Methods section.

The ether functional group is expected to affect the rate constant, which can be investigated by comparing reactions occurring on the same or opposite sides of the functional group and with the same ring size, peroxy, and radical positions. Examples are shown in Figure 3. The relative position of the ether functional group to the reactive sites (peroxy and radical) can be classified as “*Out*”, where the two reactive sites are on the same side of the ether group and therefore “outside” the transition state ring, or “*In*” where the reactive sites are on opposite sides of the ether group and thus “inside” the transition state ring. To examine the effect of the ether functional group, we compared the *In* and *Out* rate constants for selected 8-membered ring reactions, as shown in Figure 4. Generally, the energy barrier of *In* and *Out* reactions barely changed for most of the selected examples. The maximum observed energy difference was 1.8 kcal/mol for 8S-T_a_ (Figure 4c), as evident by the differences in rate constant slopes, resulting in approximately an order of magnitude higher rate constant for 8S-T_a_ Out at 500 K. On the other hand, the entropy effect resulted in differences slightly above an order of magnitude, as in 8S_a_-T (Figure 4d). This can be partially attributed to the differences in the HR barriers, as relatively higher barriers were observed for C–O rotors compared to C–C rotors, as shown in the Supporting Information. Locking the C–O rotors within the *In* transition state results in higher rotational barriers and entropy only for the reactant, and hence higher rate constants for the *In* reaction. On the other hand, the high C–O rotational barrier in the *Out* reactant is offset by the rotational barrier of the same C–O bonds present in the *Out* transition state. Therefore, unless the disparities in the energy barrier govern the rate constant differences, we anticipate higher rate constants for the *In* rate constants compared to the *Out*. Exceptions arise when the C–O barrier heights in the reactant and transition state are not equivalent, leading to *Out* > *In*, as observed in the case of 8S–S_a_ (Figure 4a). While the *Out* barrier height is 0.7 kcal/mol lower, contributing to the faster rate constant, the slight difference noted at high temperature is an entropy effect. It is noteworthy that the rotational barriers for the 7- and 8-membered rings were assigned in analogy to the calculated 5- and 6-membered ring rotational barriers; hence, inaccuracies may potentially affect the results.

### 3.2 Effect of the Peroxy and Abstracted Radical Position on Rate Constants

**Figure 5:**
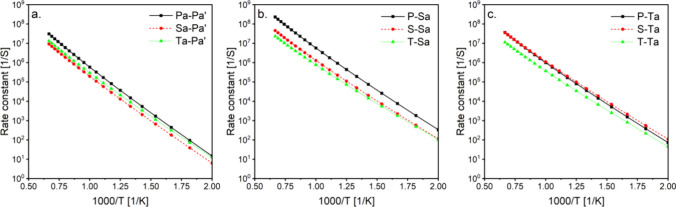
Effect of peroxy group position on 6-membered ring calculated rate constants. Isomerization nomenclature is explained in the Methods section.

**Figure 6:**
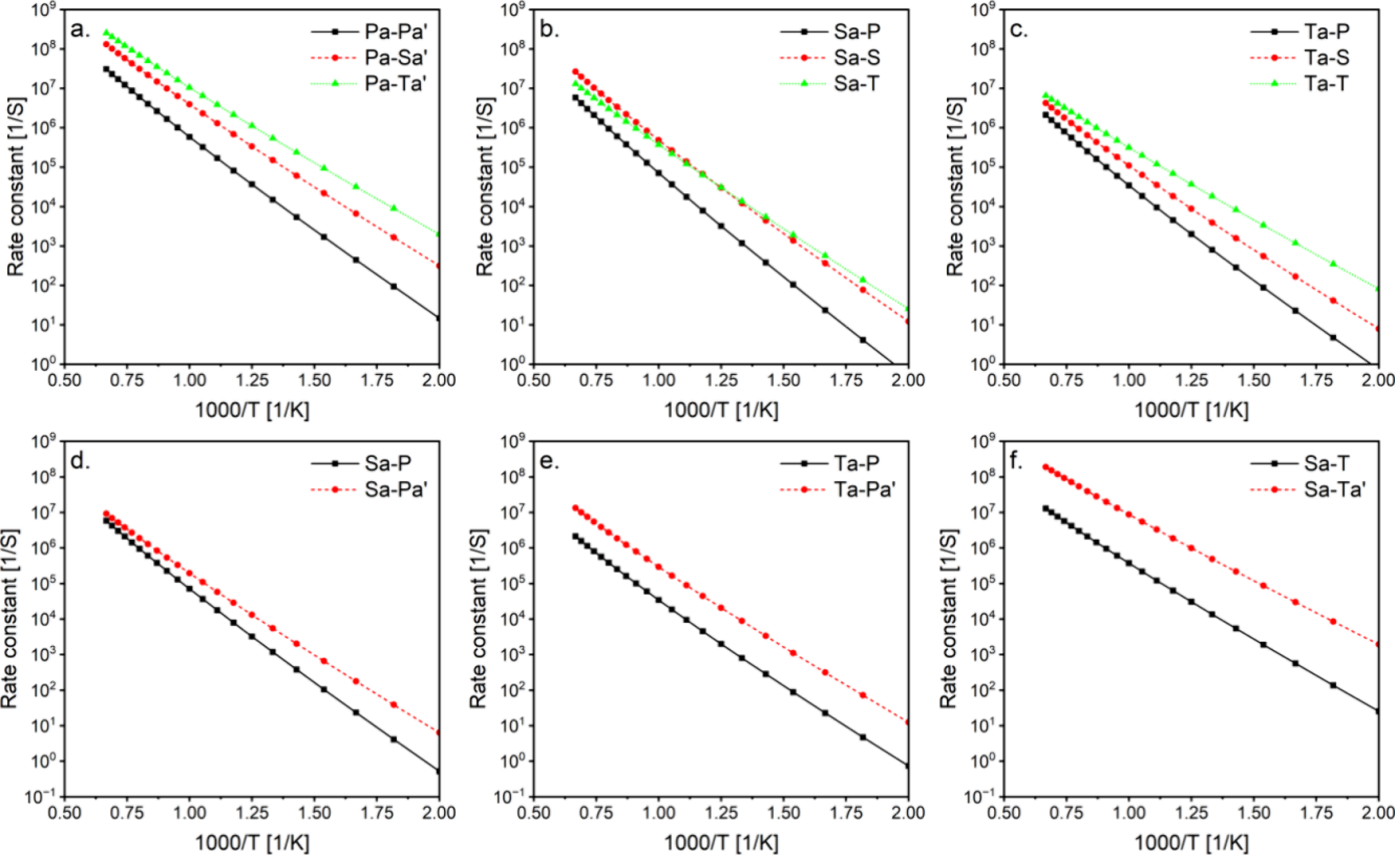
Effect of abstracted radical position on 6-membered ring calculated rate constants. (a–c) comparing abstraction from primary, secondary, and tertiary carbons. (d–f) Comparing abstraction from alpha and nonalpha carbons to the ether functional group. Isomerization nomenclature is explained in the Methods section.

To understand the impact of the peroxy radical and the abstracted radical positions, the rate constants for various 6-membered ring were compared as shown in Figures 5 and 6. In Figure 5, the position of the peroxy group was varied (primary, secondary, and tertiary) while maintaining the same abstracted radical position. Generally, the rate constants were similar to slight faster rates observed for primary peroxy groups. Only minor differences were noted at low temperatures, where the maximum energy difference between the compared rates was 1.1 kcal/mol. At high temperatures, entropy effects led to differences of typically half an order of magnitude, with an order of magnitude difference observed in Figure 5b. Changing the position of the peroxy group (P(OO), S(OO), or T(OO)) alters the nature of the rotors neighboring the peroxy group, thereby influencing the interactions. Higher substitution of the peroxy carbon (S(OO) or T(OO)) results in more gauche interactions and chirality, which affects the rotational barriers and entropy.^33^

On the other hand, the effect of the abstracted radical position (primary, secondary, or tertiary) was governed by the barrier differences as significant variations are observed at low temperatures in Figure 6a–c. These variations resulted from energy differences ranging from 4.3 to 5.3 kcal/mol between the fastest (abstraction to form tertiary radical) and slowest (abstraction to form primary radical) rate constants. The rate constants followed the trend of faster rates for abstractions from tertiary > secondary > primary^34^ aligning with their bond dissociation energies (BDE).

Figure 6.d-f also illustrates the impact of the abstracted radical being either alpha or nonalpha to the ether functional group, with faster rate constants observed for the former. The ether functional group weakens the alpha C–H BDE while strengthening the beta C–H BDE.^2^ This results in relatively lower energy barriers for abstractions from alpha sites by, 3.5, 2.1, and 2.6 kcal/mol for the cases shown in Figure 6d–f, respectively. This is also evident from the barrier heights reported in Table 1, where abstractions from alpha carbons show lower barrier heights within each group of reactions categorized by ring size. Entropy also contributes to the observed differences, particularly at high temperatures, which can be explained by the fact that each compared pair of reactions in Figure 6d–f, undergoes isomerization via *In* or *Out* transition states. The varying rotational barriers associated with the *In* and *Out* cases may influence the entropy, as discussed in the previous section.

### 3.3 Comparison with the Literature

Our G4 calculated rate constants were compared to the literature rate constants, which were determined using different levels of theory, including CCSD(T)/aug-cc-pVTZ//M06-2X/cc-pVTZ,^19^ CCSD(T)-F12b/cc-pVTZ-F12//B2PLYPD3/cc-pVTZ,^16^ CBS-QB3//B3LYP/CBSB7,^15^ and CCSD(T)-F12a/jun-cc-pVDZ//M05-2X/jun-cc-pVTZ.^12^ These studies addressed the isomerization of DEE, and therefore are limited to 6 isomerization reactions, compared to the more than 120 site-specific rate constants determined in our study.

**Figure 7:**
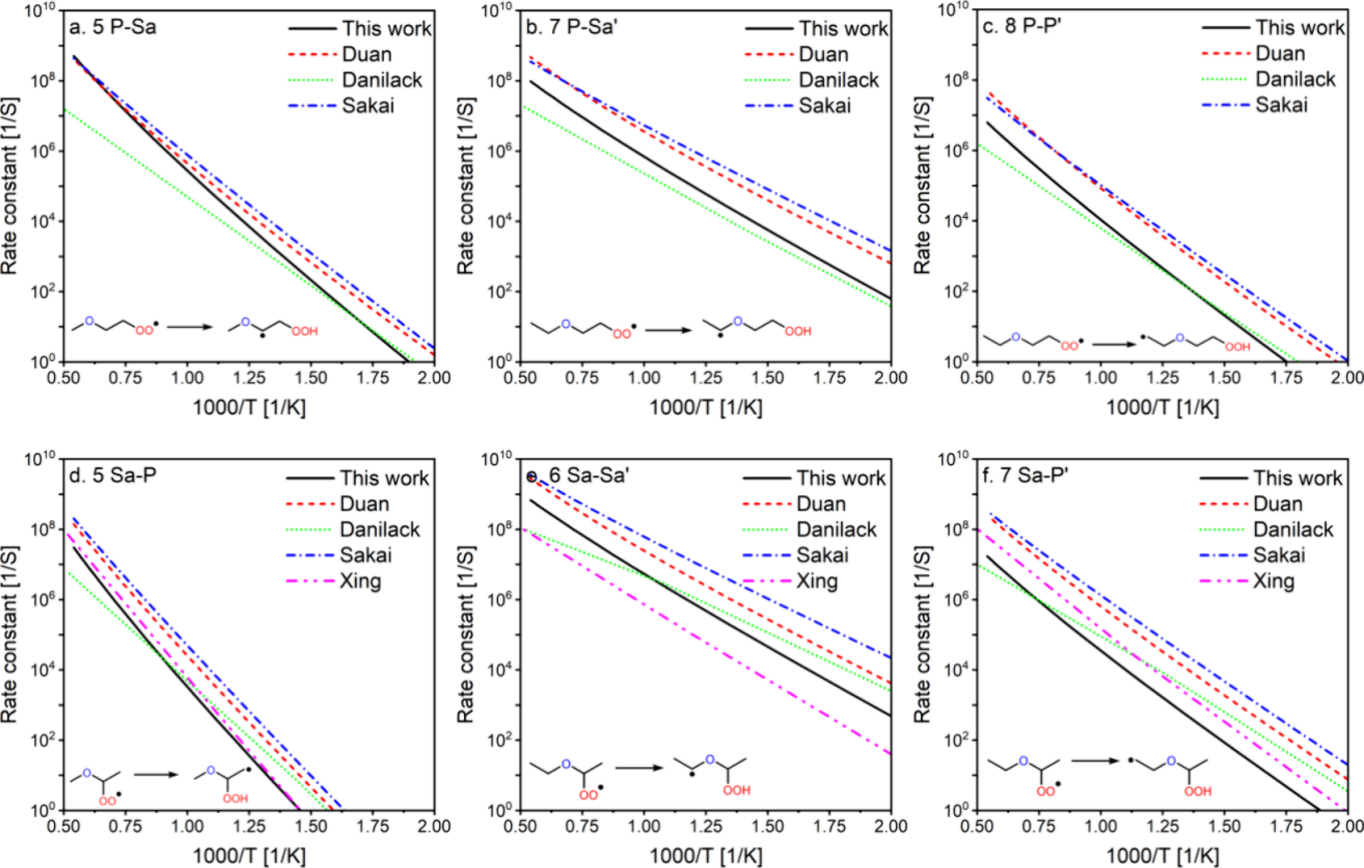
Calculated rates in this study (black solid lines) compared to literature values from Duan et al.,^19^ Danilack et al.,^16^ Sakai et al.,^15^ and Xing et al.^12^ Isomerization nomenclature is explained in the Methods section.

Generally, our calculated rate constants showed similar temperature dependence to Duan et al.^19^ and Xing et al.,^12^ but a different temperature dependence compared to Danilack et al.^16^ and Sakai et al.^15^ in a few cases, as shown in Figure 7. This suggests that the energy effect plays a major role in the discrepancy with the latter. Our calculated rate constants agreed well with the master equation coupled cluster rate constants reported by Danilack et al.,^16^ especially at low temperatures. However, our rate constants were generally slower than those reported by Duan et al.^19^ and Sakai et al.,^15^ primarily due to the entropy effect, except for 5P–S_a_ (Figure 7a), where we observed an order of magnitude difference at low temperature (500 K), likely due to differences in barrier heights. Duan et al.^19^ used the multistructural anharmonicity treatment, which reduced the variational rate constants. They indicated that neglecting multistructural anharmonicity effects may lead to overestimated rate constants by up to an order of magnitude. However, although we did not include multistructural torsional anharmonicity in our calculations and only used the 1D HR treatment, we still obtained slower rate constants than Duan et al.^19^ Moreover, Duan et al.^19^ suggested that the underestimation of barrier heights, the 1D HR treatment, and neglecting multistructural torsional anharmonicity, in addition to the transition state theory methods adopted by Sakai et al.,^15^ resulted in their slightly faster rate constants. Interestingly, our study is very similar to Sakai et al.’s work in these respects,^15^ but we obtained slower rate constants, implying that differences in energy may be the major reason for the observed discrepancies.

### 3.4 Comparison with the Alkane Rate Constants

**Figure 8:**
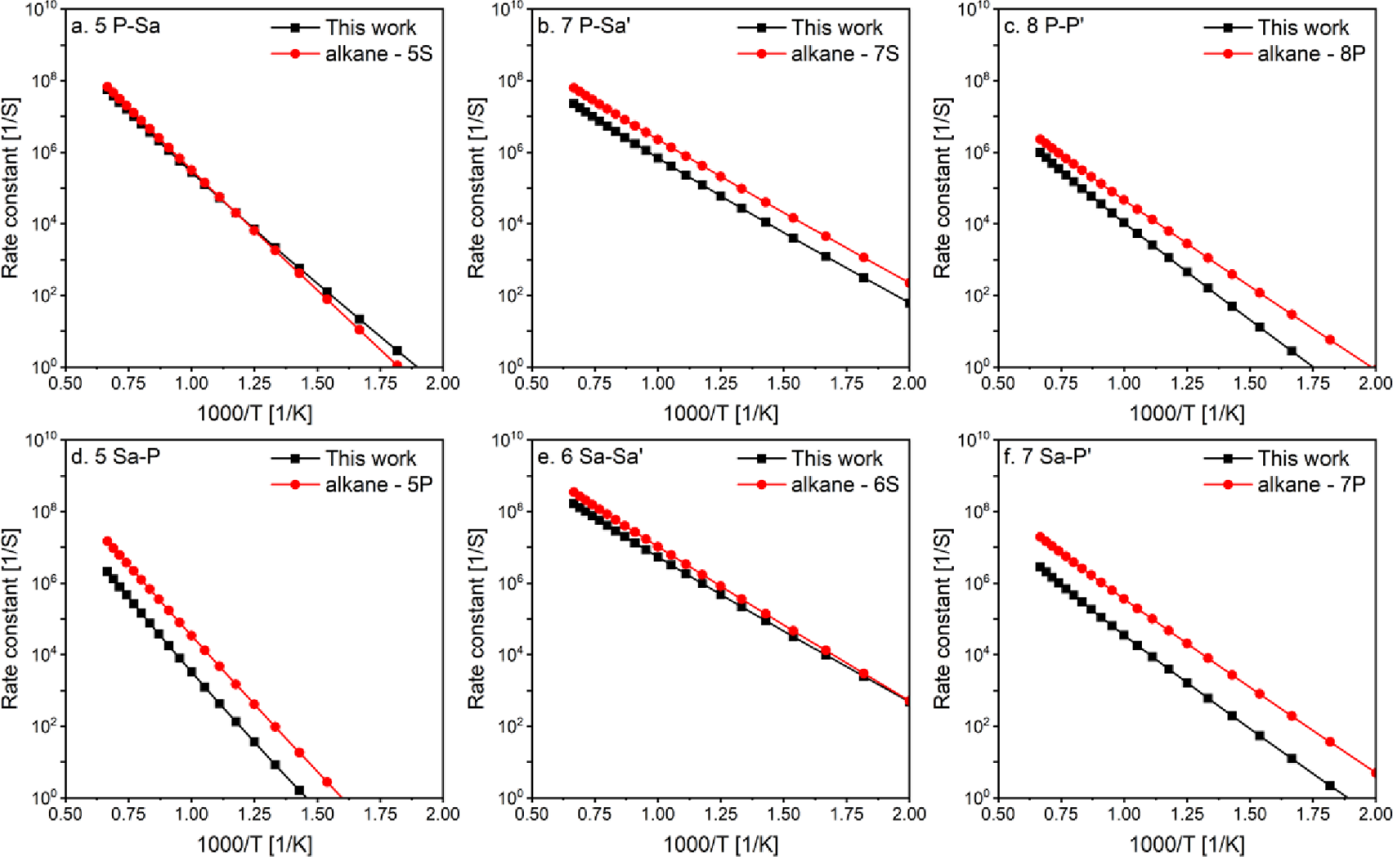
Calculated rates in this study (black lines) compared to analogous alkane rate constants (red lines). Isomerization nomenclature is explained in the Methods section.

We also compared our rate constants to the alkane isomerization rate constants, typically adopted by analogy when site-specific rate constants are unavailable. Figure 8 shows these rate constants are similar or higher than our calculated ether rate constants. This could result in overestimating rate constants and branching ratios for the ether RO_2_ isomerization reactions. The differences can exceed an order of magnitude at low temperatures (500 K), highlighting the need for site-specific rate constants when available. The differences can be attributed to the entropic term, where significant differences are observed at high temperatures, such as in the case of 5S_a_-P and 7S_a_-P′ (Figures 8d,f, respectively). In some cases, the energy barrier contributes to the observed differences, such as in 8P–P′, where more than an order of magnitude difference is detected at low temperatures (500 K).

### 3.5 Effect on Di-Iso-Butyl Ether Kinetic Models

**Figure 9:**
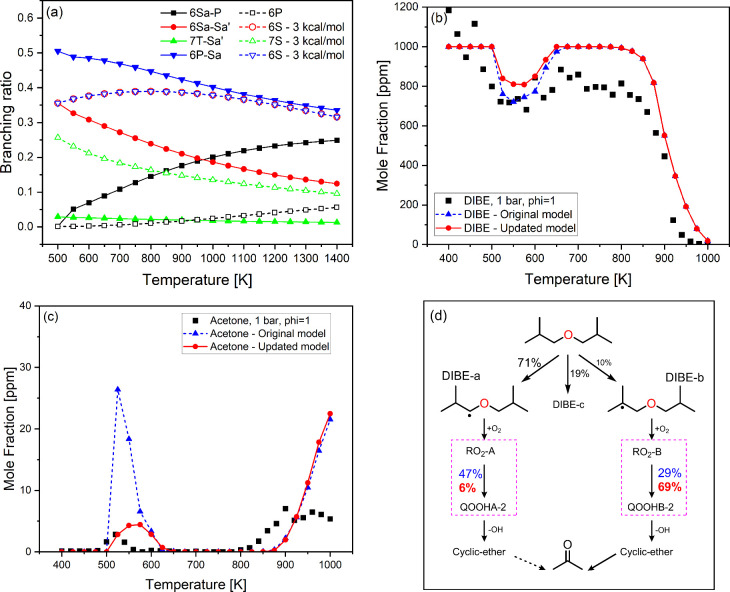
(a) Branching ratios for RO_2_ isomerization using the calculated ether’s site-specific rate constants (solid lines) and the original rate constants analogous to alkane rate constants^36^ (dashed lines). (b) and (c) Flow reactor speciation for DIBE and acetone, respectively, using ether’s site-specific rate constants (solid red lines) and alkane rate constants^36^ (dashed blue lines). (d) DIBE reaction pathways leading to acetone, blue and red fluxes are calculated using the original and updated models, respectively.

To evaluate the effect of the calculated rate constants, we tested them in di-iso-butyl ether (DIBE) mechanisms developed in ref (35) (original model). We initially used RO_2_ isomerization rate constants in analogy to alkane rate constants from Villano et al.,^36^ with activation energy reduced by 3 kcal/mol for reactions where abstracted hydrogen is alpha to the ether functional group, similar to the di-*n*-butyl ether mechanism in the literature.^8,37^ We updated the RO_2_ isomerization rate constants in the mechanism (updated model) using our calculated rate constants and compared them to the original model. Figure 9a shows the RO_2_ branching ratio for the major pathways of RO_2_ isomerization in DIBE. The branching ratio is calculated as the ratio of a site-specific isomerization rate constant to the total RO_2_ isomerization rate constants at the same temperature. Using alkane rate rules on the original mechanism resulted in an overestimated branching ratio for 6S_a_–S_a_′ and 7T–S_a_′ by a factor of 1.5 (at 700 K) and 9 (at 500 K), respectively, and underestimated branching ratio for 6S_a_–P and 6P–S_a_ by a factor of 35 and 1.3, respectively, at 550 K. It is important to note that alkane rate constants cannot distinguish between secondary sites and alpha secondary (S_a_) to the ether functional group. Alkane rates are also independent of the chemical nature of the carbons where the peroxy radical is located, resulting in the same rate constants and branching ratios for 6S_a_–S_a_′ and 6P–S_a_ (identical branching ratios in Figure 9a). However, using our calculated rate constants for these pathways led to branching ratios that differ by approximately a factor of 2 at 700 K.

The effect of the new rate constants and the resulting differences in branching ratios was also studied by comparing the species profile of DIBE in flow reactor experiments at 1 bar and phi = 1^35^ using the original and updated DIBE kinetic model. As depicted in Figure 9b,c, the conversion or reactivity of DIBE is decreased at the negative temperature coefficient (NTC) region, and a better agreement for the acetone prediction with the experimental results was observed. This can be attributed to differences in the dominant site-specific pathways of RO_2_ isomerization, and therefore the subsequent chemistry. We conducted flux analysis at 1 bar and 550 K using both the original and updated model (Figure 9d) to understand acetone production pathways and the underlying chemistry responsible for the observed differences in predictions. At these conditions, DIBE oxidizes to primarily form DIBE-A radical (71%), followed by DIBE-c (19%) and DIBE-a (10%) radicals, which undergo typical low temperature chemistry. Acetone was predominantly produced from cyclic ether intermediates, particularly those formed form QOOHA-2 and QOOHB-2 radicals. Updating the rate constant of RO_2_ isomerization reduced the flux of RO_2_-A to QOOHA-2 from 47 to 6%, while increased the flux of RO_2_–B to QOOHB-2 from 29 to 69%. This shifted the acetone formation pathway from the highly produced QOOHA-2 to QOOHB-2 after the model updates. The latter QOOHB-2 radical is formed from DIBE-b which represents only 10% of DIBE leading to the observed decrease in acetone production.

Updating certain reaction pathways in the kinetic model likely results in an initial model that shows discrepancies with experimental data, as altering the rate constants for one class of reaction necessitates tuning or updating the rate constants for other classes of reactions. Therefore, it is imperative to update or calculate site-specific rate constants for other classes of reactions, such as cyclic ether formation and second isomerization. Accurate thermodynamic properties are also essential for model accuracy and may contribute to discrepancies between simulations and experiment predictions, as the reverse rate of these reactions is primarily determined using thermodynamic data.

## 4 Conclusions

This work thoroughly investigated the kinetics of alkylperoxy radical (RO_2_) isomerizations for ethers. Rate parameters were calculated for 5-, 6-, 7-, and 8-membered ring transition states, with all possible reactive sites (peroxy and abstracted sites). The obtained rate constants were compared to existing rates in the literature for DEE, which generally showed reasonable agreement. Furthermore, rate constants were compared to alkane rate constants to highlight the effect of applying analogous rate constants in ether combustion chemistry. This was examined by DIBE RO_2_ isomerization branching ratio and flow reactor speciation, where using the new rate constants resulted in different branching ratios and fuel conversion. This study is the first systematic study to provide RO_2_ isomerization rate constants for ethers and emphasizes the significance of using accurate site-specific rate constants for reliable combustion modeling of ethers. Additionally, the study underscores the importance of further exploring site-specific rate constants for other low-temperature reactions for diesel-boiling-range ethers.

## Supporting Information Available

Isodesmic reactions for selected species, rotational barriers for selected species, estimated enthalpies of formation at 298 K for reactants, products and transition states, and list of all calculated reactions (PDF)
