# Supplementary material for: High-Pressure Rate Rules for Ether Alkylperoxy Radical Isomerization
Source: J Phys Chem A. 2024 Sep 17;128(38):8220–31. doi: 10.1021/acs.jpca.4c04840 (PMC11440582; doi:10.1021/acs.jpca.4c04840)
Supplement: Supplementary file 1 — jp4c04840_si_001.pdf [file jp4c04840_si_001.pdf]

# High-Pressure Rate Rules for Ether Alkylperoxy Radical Isomerization

Samah Y. Mohamed[1], Yeonjoon Kim[2], Gina Fioroni[1], Seonah Kim[1,2], and Robert McCormick[1]

[1]*National Renewable Energy Laboratory, Golden, CO 80401 United States*

[2]*Chemistry Department, Colorado State University, Fort Collins, CO 80523 United States*

**Table S 1: Comparisons of isodesmic and atomization energy in kcal/mol for selected species** ....1
**Table S 2: Rotational barrier 8Sa-T reactants and transition state rotors** ....2
**Table S 3: Enthalpies of formation and barriers for all reactants, transition states, and products in kcal/mol** ....3
**Table S 4: complete list of the calculated RO2 reactants** ....8

**Table S 1: Comparisons of isodesmic and atomization energy in kcal/mol for selected species**

| Reaction | Isodesmic energy | Atomization energy | Energy difference (Δ) |
|---|---|---|---|
| 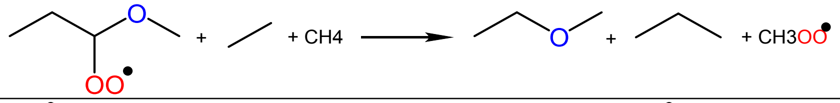 | −52.3 | −53.2 | −0.8 |
| 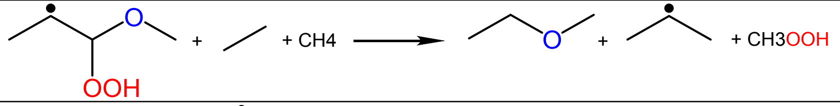 | −38.0 | −39.2 | −1.2 |
| 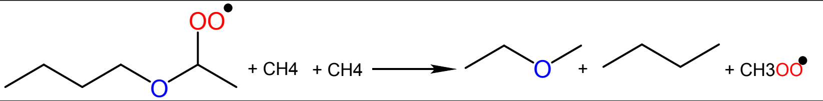 | −66.5 | −67.4 | −0.9 |
| 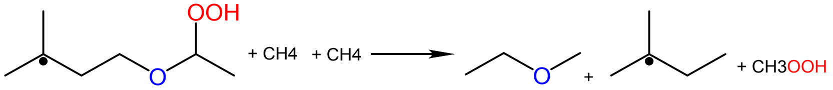 | −64.9 | −64.4 | 0.5 |

**Table S 2: Rotational barrier 8Sa-T reactants and transition state rotors**

<table>
<tr><th colspan="2">8S<sub>a</sub>-T’ In</th><th colspan="2">8S<sub>a</sub>-T Out</th></tr>
<tr><td>Reactant<br>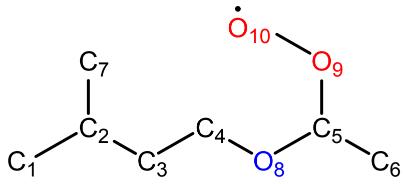</td><td>Transition state (TS)<br>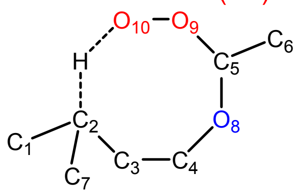</td><td>Reactant<br>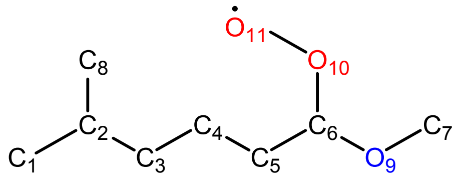</td><td>Transition state (TS)<br>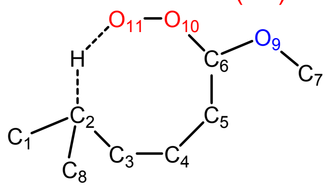</td></tr>
<tr><td colspan="2">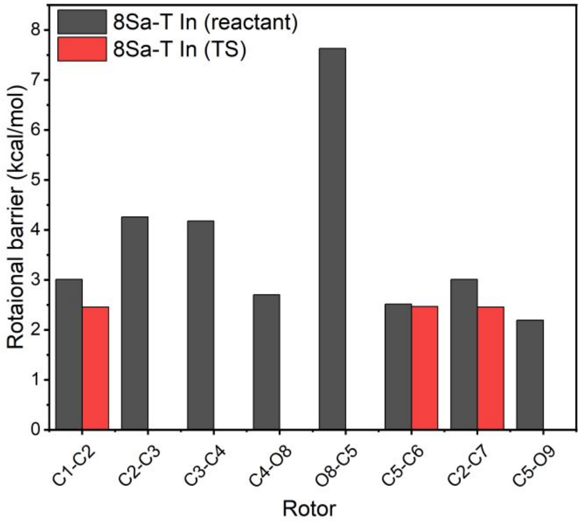
</td><td colspan="2">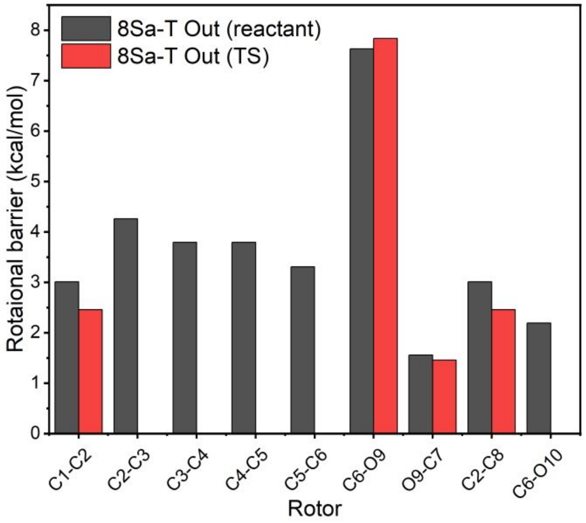
</td></tr>
</table>

## Table S 3: Enthalpies of formation and barriers for all reactants, transition states, and products in kcal/mol

| Reaction | (OO)-Rad* | $\Delta H_f$(298K) [kcal/mol] | | | Barrier [kcal/mol] |
|---|---|---|---|---|---|
| | | Reactants | Products | Transition states | |
| **5 membered ring (N=5)** | | | | | |
| [O]OCCOC → CO[CH]COO | P-$S_a$ | -37.4 | -28.6 | -7.7 | 29.70 |
| [O]OCC(C)OC → CO[C](C)COO | P-$T_a$ | -46.4 | -37.7 | -20.1 | 26.27 |
| [O]OC(OC)C → [CH2]C(OC)OO | $S_a$-P | -47.8 | -30.0 | -11.8 | 36.03 |
| [O]OC(CC)OC → C[CH]C(OC)OO | $S_a$-S | -53.2 | -39.2 | -20.5 | 32.65 |
| [O]OC(C)COC → CO[CH]C(C)OO | S-$S_a$ | -47.3 | -38.0 | -19.1 | 28.20 |
| [O]OC(C)C(C)OC → CO[C](C)C(C)OO | S-$T_a$ | -56.1 | -46.2 | -30.0 | 26.03 |
| [O]OC(OC)C(C)C → COC(OO)[C](C)C | $S_a$-T | -60.4 | -49.5 | -30.8 | 29.63 |
| [O]OC(C)(OC)C → [CH2]C(C)(OC)OO | $T_a$-P | -57.1 | -41.0 | -22.5 | 34.57 |
| [O]OC(C)(C)COC → CO[CH]C(C)(C)OO | T-$S_a$ | -58.0 | -47.3 | -29.6 | 28.43 |
| [O]OC(OC)(C)CC → C[CH]C(C)(OC)OO | $T_a$-S | -62.5 | -50.3 | -31.6 | 30.94 |
| [O]OC(C)(OC)C(C)C → COC(C)(OO)[C](C)C | $T_a$-T | -69.4 | -58.9 | -41.5 | 27.94 |
| [O]OC(C)(C)C(C)OC → CO[C](C)C(C)(C)OO | T-$T_a$ | -66.3 | -55.6 | -39.4 | 26.83 |
| **6 membered ring (N=6)** | | | | | |
| COCO[O] → [CH2]OCOO | $P_a$-$P_a$' | -35.6 | -26.5 | -14.3 | 21.32 |
| CCOCO[O] → C[CH]OCOO | $P_a$-$S_a$' | -44.3 | -36.4 | -25.0 | 19.28 |
| COCCCO[O] → CO[CH]CCOO | P-$S_a$ | -45.1 | -36.3 | -24.5 | 20.57 |
| CC(C)OCO[O] → C[C](C)OCOO | $P_a$-$T_a$' | -53.0 | -45.2 | -35.5 | 17.56 |
| COC(C)CCO[O] → CO[C](C)CCOO | P-$T_a$ | -54.1 | -45.5 | -34.1 | 19.94 |
| CCC(OC)O[O] → [CH2]CC(OC)OO | $S_a$-P | -53.2 | -37.4 | -28.7 | 24.47 |

| | | | | | |
|---|---|---|---|---|---|
| COC(C)O[O] → [CH2]OC(C)OO | $S_a$-$P_a$' | -47.8 | -37.3 | -26.9 | 20.97 |
| COCCC(C)O[O] → CO[CH]CC(C)OO | S-$S_a$ | -55.0 | -46.1 | -34.9 | 20.09 |
| COC(O[O])CCC → C[CH]CC(OC)OO | $S_a$-S | -58.7 | -45.5 | -36.5 | 22.16 |
| CC(O[O])OCC → C[CH]OC(C)OO | $S_a$-$S_a$' | -56.5 | -47.1 | -37.5 | 18.97 |
| COC(C)CC(C)O[O] → CO[C](C)CC(C)OO | S-$T_a$ | -64.0 | -55.0 | -44.5 | 19.51 |
| COC(O[O])CC(C)C → COC(C[C](C)C)OO | $S_a$-T | -65.9 | -55.0 | -46.0 | 19.91 |
| CC(C)OC(C)O[O] → C[C](C)OC(C)OO | $S_a$-$T_a$' | -65.3 | -55.1 | -48.0 | 17.33 |
| COC(C)(O[O])CC → COC(C)(OO)C[CH2] | $T_a$-P | -62.5 | -49.0 | -39.6 | 22.97 |
| COC(C)(C)O[O] → [CH2]OC(C)(C)OO | $T_a$-$P_a$' | -57.1 | -48.3 | -36.3 | 20.81 |
| COCCC(C)(C)O[O] → CO[CH]CC(C)(C)OO | T-$S_a$ | -64.2 | -56.8 | -44.4 | 19.83 |
| COC(C)(O[O])CCC → C[CH]CC(C)(OC)OO | $T_a$-S | -68.0 | -57.1 | -47.3 | 20.70 |
| CCOC(C)(C)O[O] → C[CH]OC(C)(C)OO | $T_a$-$S_a$' | -65.9 | -58.0 | -46.9 | 18.92 |
| COC(C)CC(C)(O[O])C → CO[C](C)CC(C)(C)OO | T-$T_a$ | -73.2 | -65.1 | -53.8 | 19.40 |
| COC(C)(O[O])CC(C)C → COC(C)(C[C](C)C)OO | $T_a$-T | -74.5 | -65.9 | -56.8 | 17.68 |
| CC(C)OC(C)(C)O[O] → C[C](C)OC(C)(C)OO | $T_a$-$T_a$' | -74.7 | -65.7 | -56.2 | 18.43 |
| **7 membered ring (N=7)** | | | | | |
| [O]OCCOC → [CH2]OCCOO | P-$P_a$’ | -37.4 | -26.7 | -15.5 | 21.89 |
| [O]OCOCC → [CH2]COCOO | $P_a$-P’ | -44.3 | -30.0 | -20.5 | 23.79 |
| [O]OCOCCC → C[CH]COCOO | $P_a$-S’ | -49.7 | -38.0 | -28.9 | 20.83 |
| [O]OCCOCC → C[CH]OCCOO | P-$S_a$' | -46.1 | -36.8 | -26.4 | 19.68 |
| [O]OCCCCOC → CO[CH]CCCOO | P-$S_a$ | -50.5 | -41.7 | -31.6 | 18.92 |
| [O]OCCOC(C)C → C[C](C)OCCOO | P-$T_a$' | -54.9 | -45.5 | -36.3 | 18.57 |
| [O]OCCCC(C)OC → CO[C](C)CCCOO | P-$T_a$ | -59.3 | -50.5 | -42.1 | 17.15 |
| [O]OCOCC(C)C → C[C](C)COCOO | $P_a$-T’ | -57.1 | -47.7 | -39.3 | 17.85 |
| [O]OC(C)OCC → [CH2]COC(C)OO | $S_a$-P' | -56.5 | -39.7 | -32.3 | 24.17 |
| [O]OC(OC)CCC → [CH2]CCC(OC)OO | $S_a$-P | -58.7 | -43.4 | -34.5 | 24.15 |
| [O]OC(C)COC → [CH2]OCC(C)OO | S-$P_a$’ | -47.3 | -36.5 | -25.5 | 21.83 |

| | | | | | |
|---|---|---|---|---|---|
| [O]OC(C)COCC → C[CH]OCC(C)OO | S-$S_a$' | -56.0 | -46.6 | -36.3 | 19.71 |
| [O]OC(C)CCCOC → CO[CH]CCC(C)OO | S-$S_a$ | -60.6 | -51.2 | -41.4 | 19.19 |
| [O]OC(C)OCCC → C[CH]COC(C)OO | $S_a$-S' | -61.9 | -48.3 | -40.6 | 21.30 |
| [O]OC(OC)CCCC → C[CH]CCC(OC)OO | $S_a$-S | -64.2 | -51.7 | -42.9 | 21.29 |
| [O]OC(C)COC(C)C → C[C](C)OCC(C)OO | S-$T_a$' | -64.9 | -55.4 | -46.3 | 18.60 |
| [O]OC(C)CCC(C)OC → CO[C](C)CCC(C)OO | S-$T_a$ | -69.4 | -60.2 | -52.0 | 17.38 |
| [O]OC(C)OCC(C)C → C[C](C)COC(C)OO | $S_a$-T' | -69.4 | -57.7 | -51.1 | 18.30 |
| [O]OC(OC)CCC(C)C → COC(CC[C](C)C)OO | $S_a$-T | -71.3 | -60.1 | -52.8 | 18.47 |
| [O]OC(C)(C)COC → [CH2]OCC(C)(C)OO | T-$P_a$' | -58.0 | -46.5 | -35.3 | 22.70 |
| [O]OC(C)(C)OCC → [CH2]COC(C)(C)OO | $T_a$-P' | -65.9 | -50.8 | -40.7 | 25.13 |
| [O]OC(OC)(C)CCC → [CH2]CCC(C)(OC)OO | $T_a$-P | -68.0 | -54.2 | -44.8 | 23.20 |
| [O]OC(C)(C)COCC → C[CH]OCC(C)(C)OO | T-$S_a$' | -66.7 | -56.0 | -46.2 | 20.50 |
| [O]OC(C)(C)CCCOC → CO[CH]CCC(C)(C)OO | T-$S_a$ | -70.6 | -62.1 | -50.6 | 20.01 |
| [O]OC(C)(C)OCCC → C[CH]COC(C)(C)OO | $T_a$-S' | -71.7 | -58.9 | -49.0 | 22.70 |
| [O]OC(OC)(C)CCCC → C[CH]CCC(C)(OC)OO | $T_a$-S | -73.7 | -62.3 | -53.3 | 20.30 |
| [O]OC(C)(C)COC(C)C → C[C](C)OCC(C)(C)OO | T-$T_a$' | -75.5 | -64.8 | -56.2 | 19.34 |
| [O]OC(C)(C)CCC(C)OC → CO[C](C)CCC(C)(C)OO | T-$T_a$ | -79.5 | -70.6 | -61.3 | 18.23 |
| [O]OC(C)(C)OCC(C)C → C[C](C)COC(C)(C)OO | $T_a$-T' | -79.3 | -68.2 | -59.6 | 19.67 |
| [O]OC(OC)(C)CCC(C)C → COC(C)(CC[C](C)C)OO | $T_a$-T | -80.7 | -71.3 | -63.0 | 17.72 |
| **8 membered ring (N=8)** | | | | | |
| [O]OCOCCC → [CH2]CCOCOO | $P_a$-P' | -49.7 | -36.7 | -27.6 | 22.06 |
| [O]OCCCOC → [CH2]OCCCOO | P-$P_a$' | -45.1 | -34.6 | -23.5 | 21.61 |
| [O]OCCCOCC → C[CH]OCCCOO | P-$S_a$' | -53.7 | -44.6 | -34.0 | 19.67 |
| [O]OCCCCCOC → CO[CH]CCCCOO | P-$S_a$ | -55.8 | -47.9 | -38.0 | 17.76 |
| [O]OCOCCCC → C[CH]CCOCOO | $P_a$-S' | -55.2 | -44.5 | -35.9 | 19.23 |
| [O]OCCCOC(C)C → C[C](C)OCCCOO | P-$T_a$' | -62.4 | -53.5 | -43.8 | 18.57 |
| [O]OCCCCC(C)OC → CO[C](C)CCCCOO | P-$T_a$ | -64.5 | -56.3 | -47.9 | 16.64 |

| | | | | | |
|---|---|---|---|---|---|
| [O]OCOCCC(C)C → C[C](C)CCOCOO | $P_a$-T' | -62.4 | -54.9 | -45.9 | 16.50 |
| [O]OC(C)OCCC → [CH2]CCOC(C)OO | $S_a$-P' | -61.9 | -46.1 | -39.2 | 22.73 |
| [O]OC(CCCC)OC → [CH2]CCCC(OC)OO | $S_a$-P | -64.2 | -48.7 | -40.8 | 23.47 |
| [O]OC(C)CCOC → [CH2]OCCC(C)OO | S-$P_a$’ | -55.0 | -44.1 | -33.3 | 21.72 |
| [O]OC(C)CCOCC → C[CH]OCCC(C)OO | S-$S_a$' | -63.6 | -52.3 | -43.8 | 19.80 |
| [O]OC(C)CCCCOC → CO[CH]CCCC(C)OO | S-$S_a$ | -65.8 | -55.0 | -46.8 | 19.07 |
| [O]OC(C)OCCCC → C[CH]CCOC(C)OO | $S_a$-S' | -67.4 | -54.5 | -47.5 | 19.90 |
| [O]OC(CCCCC)OC → C[CH]CCCC(OC)OO | $S_a$-S | -69.8 | -56.8 | -49.1 | 20.61 |
| [O]OC(C)CCOC(C)C → C[C](C)OCCC(C)OO | S-$T_a$' | -72.4 | -62.3 | -53.7 | 18.69 |
| [O]OC(C)CCCC(C)OC → CO[C](C)CCCC(C)OO | S-$T_a$ | -74.6 | -65.7 | -57.7 | 16.94 |
| [O]OC(C)OCCC(C)C → C[C](C)CCOC(C)OO | $S_a$-T' | -74.7 | -64.4 | -57.5 | 17.18 |
| [O]OC(CCCC(C)C)OC → COC(CCC[C](C)C)OO | $S_a$-T | -76.8 | -65.7 | -59.1 | 17.71 |
| [O]OC(C)(C)OCCC → [CH2]CCOC(C)(C)OO | $T_a$-P' | -71.7 | -58.1 | -50.0 | 21.74 |
| [O]OC(C)(CCCC)OC → [CH2]CCCC(C)(OC)OO | $T_a$-P | -73.7 | -59.9 | -50.9 | 22.76 |
| [O]OC(C)(C)CCOC → [CH2]OCCC(C)(C)OO | T-$P_a$' | -64.2 | -54.6 | -43.8 | 20.38 |
| [O]OC(C)(C)CCOCC → C[CH]OCCC(C)(C)OO | T-$S_a$' | -72.8 | -64.6 | -54.4 | 18.46 |
| [O]OC(C)(C)CCCCOC → CO[CH]CCCC(C)(C)OO | T-$S_a$ | -76.0 | -66.9 | -58.3 | 17.68 |
| [O]OC(C)(C)OCCCC → C[CH]CCOC(C)(C)OO | $T_a$-S' | -77.3 | -66.7 | -58.3 | 18.95 |
| [O]OC(C)(CCCCC)OC → C[CH]CCCC(C)(OC)OO | $T_a$-S | -79.2 | -68.0 | -59.3 | 19.91 |
| [O]OC(C)(C)CCOC(C)C → C[C](C)OCCC(C)(C)OO | T-$T_a$' | -81.5 | -73.5 | -64.3 | 17.15 |
| [O]OC(C)(C)CCCC(C)OC → CO[C](C)CCCC(C)(C)OO | T-$T_a$ | -84.8 | -76.4 | -68.3 | 16.48 |
| [O]OC(C)(C)OCCC(C)C → C[C](C)CCOC(C)(C)OO | $T_a$-T' | -84.9 | -75.4 | -68.4 | 16.49 |
| [O]OC(C)(CCCC(C)C)OC → COC(C)(CCC[C](C)C)OO | $T_a$-T | -86.3 | -76.9 | -69.3 | 17.01 |
| [O]OCCOCC → [CH2]COCCOO | P-P' | -46.1 | -30.2 | -20.7 | 25.33 |
| [O]OCCOCCC → C[CH]COCCOO | P-S' | -51.5 | -38.2 | -28.9 | 22.56 |
| [O]OCCOCC(C)C → C[C](C)COCCOO | P-T' | -59.0 | -47.6 | -39.6 | 19.40 |
| [O]OC(C)COCC → [CH2]COCC(C)OO | S-P' | -56.0 | -40.0 | -30.9 | 25.12 |

| | | | | | |
|---|---|---|---|---|---|
| [O]OC(C)COCCC → C[CH]COCC(C)OO | S-S' | -61.5 | -48.0 | -39.1 | 22.38 |
| [O]OC(C)COCC(C)C → C[C](C)COCC(C)OO | S-T' | -69.0 | -57.4 | -49.8 | 19.15 |
| [O]OC(C)(C)COCC → [CH2]COCC(C)(C)OO | T-P' | -66.7 | -49.4 | -41.8 | 24.88 |
| [O]OC(C)(C)COCCC → C[CH]COCC(C)(C)OO | T-S' | -72.2 | -57.3 | -50.0 | 22.13 |
| [O]OC(C)(C)COCC(C)C → C[C](C)COCC(C)(C)OO | T-T' | -79.7 | -66.9 | -60.9 | 18.81 |

**Table S 4: complete list of the calculated RO2 reactants**

| Reaction | (OO)-Rad | |
|---|---|---|
| | **5-membered ring (N = 5)** | |
| [O]OCCOC → CO[CH]COO | P-Sa | 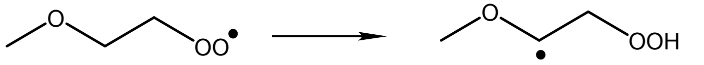 |
| [O]OCC(C)OC → CO[C](C)COO | P-Ta | 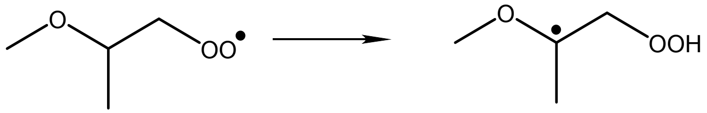 |
| [O]OC(OC)C → [CH2]C(OC)OO | Sa-P | 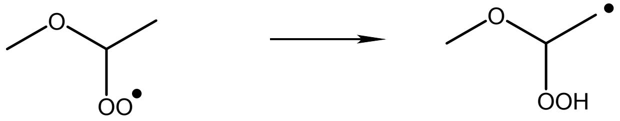 |
| [O]OC(CC)OC → C[CH]C(OC)OO | Sa-S | 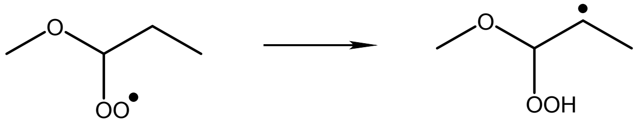 |
| [O]OC(C)COC → CO[CH]C(C)OO | S-Sa | 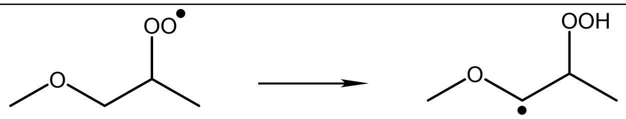 |
| [O]OC(C)C(C)OC → CO[C](C)C(C)OO | S-Ta | 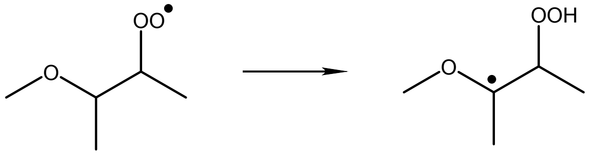 |
| [O]OC(OC)C(C)C → COC(OO)[C](C)C | Sa-T | 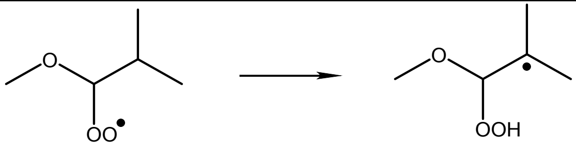 |

| | | |
|---|---|---|
| [O]OC(C)(OC)C → [CH2]C(C)(OC)OO | Ta-P | 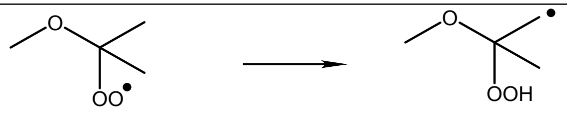 |
| [O]OC(C)(C)COC → CO[CH]C(C)(C)OO | T-Sa | 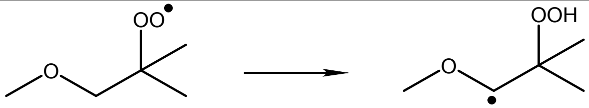 |
| [O]OC(OC)(C)CC → C[CH]C(C)(OC)OO | Ta-S | 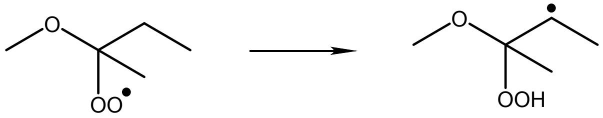 |
| [O]OC(C)(OC)C(C)C → COC(C)(OO)[C](C)C | Ta-T | 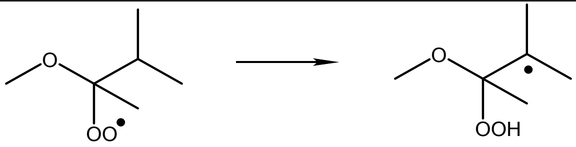 |
| [O]OC(C)(C)C(C)OC → CO[C](C)C(C)(C)OO | T-Ta | 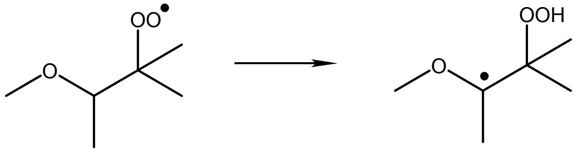 |
| **6-membered ring (N = 6)** | | |
| COCO[O] → [CH2]OCOO | Pa-Pa' | 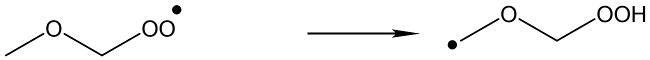 |
| CCOCO[O] → C[CH]OCOO | Pa-Sa' | 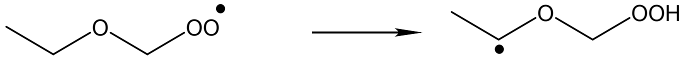 |
| COCCCO[O] → CO[CH]CCOO | P-Sa | 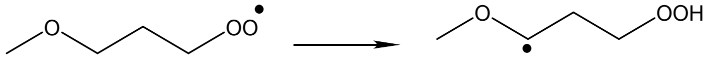 |
| CC(C)OCO[O] → C[C](C)OCOO | Pa-Ta' | 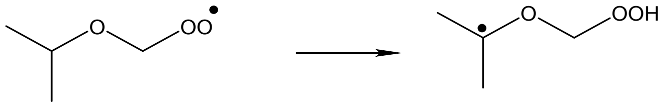 |

| | | |
|---|---|---|
| COC(C)CCO[O] → CO[C](C)CCOO | P-Ta | 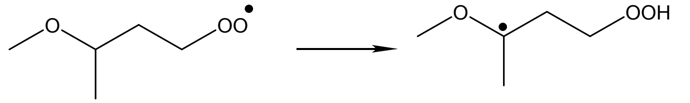 |
| CCC(OC)O[O] → [CH2]CC(OC)OO | Sa-P | 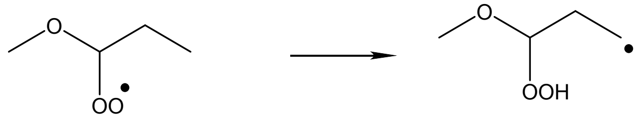 |
| COC(C)O[O] → [CH2]OC(C)OO | Sa-Pa' | 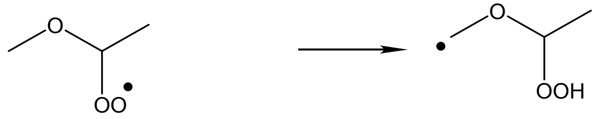 |
| COCCC(C)O[O] → CO[CH]CC(C)OO | S-Sa | 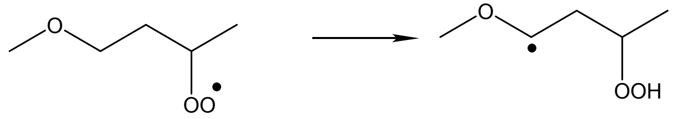 |
| COC(O[O])CCC → C[CH]CC(OC)OO | Sa-S | 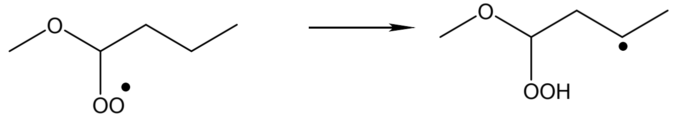 |
| CC(O[O])OCC → C[CH]OC(C)OO | Sa-Sa' | 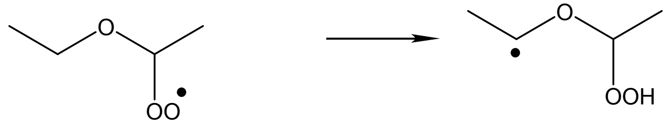 |
| COC(C)CC(C)O[O] → CO[C](C)CC(C)OO | S-Ta | 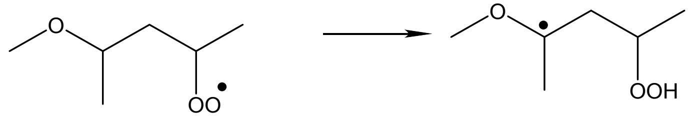 |
| COC(O[O])CC(C)C → COC(C[C](C)C)OO | Sa-T | 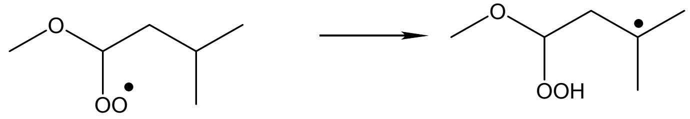 |

| | | |
|---|---|---|
| CC(C)OC(C)O[O] → C[C](C)OC(C)OO | Sa-Ta' | 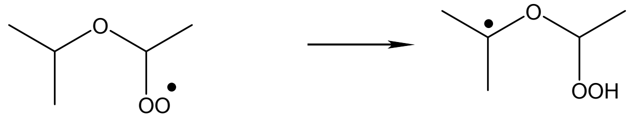 |
| COC(C)(O[O])CC → COC(C)(OO)C[CH2] | Ta-P | 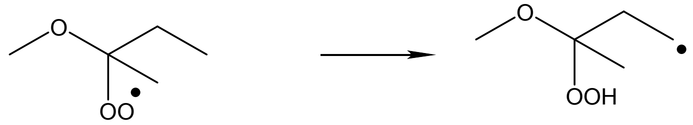 |
| COC(C)(C)O[O] → [CH2]OC(C)(C)OO | Ta-Pa' | 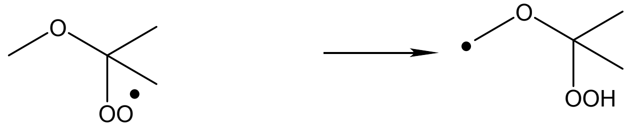 |
| COCCC(C)(C)O[O] → CO[CH]CC(C)(C)OO | T-Sa | 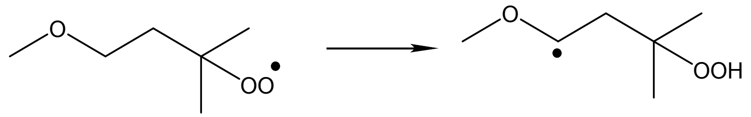 |
| COC(C)(O[O])CCC → C[CH]CC(C)(OC)OO | Ta-S | 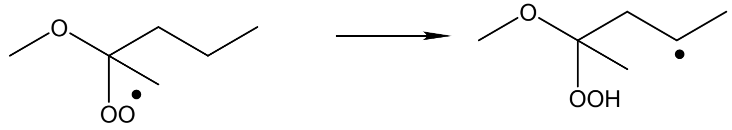 |
| CCOC(C)(C)O[O] → C[CH]OC(C)(C)OO | Ta-Sa' | 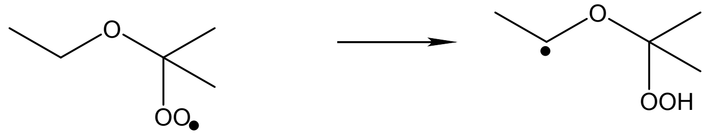 |
| COC(C)CC(C)(O[O])C → CO[C](C)CC(C)(C)OO | T-Ta | 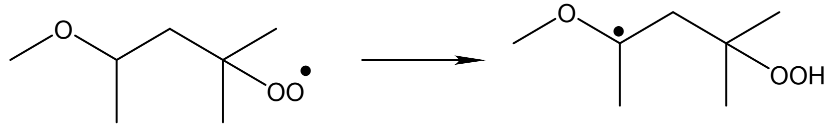 |
| COC(C)(O[O])CC(C)C → COC(C)(C[C](C)C)OO | Ta-T | 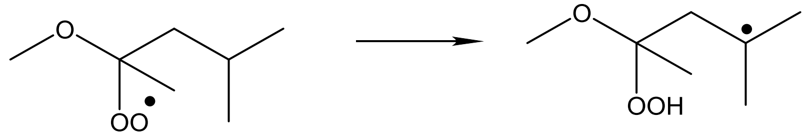 |

| | | |
|---|---|---|
| CC(C)OC(C)(C)O[O] → C[C](C)OC(C)(C)OO | Ta-Ta' | 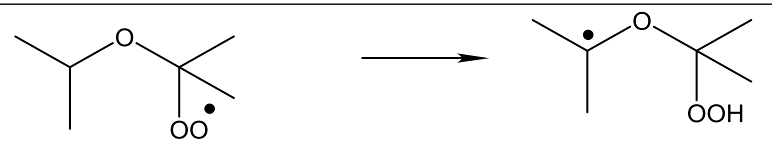 |
| **7-membered ring (N = 7)** | | |
| [O]OCCOC → [CH2]OCCOO | P-Pa’ | 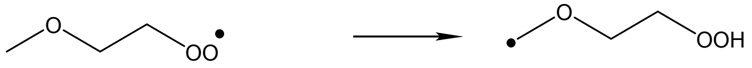 |
| [O]OCOCC → [CH2]COCOO | Pa-P’ | 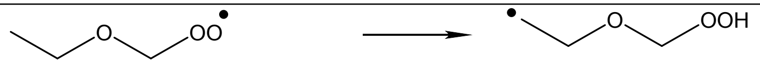 |
| [O]OCOCCC → C[CH]COCOO | Pa-S’ | 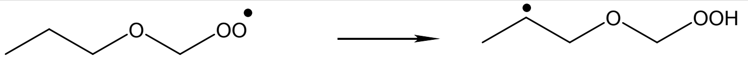 |
| [O]OCCOCC → C[CH]OCCOO | P-Sa' | 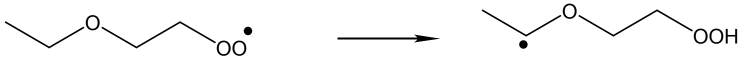 |
| [O]OCCCCOC → CO[CH]CCCOO | P-Sa | 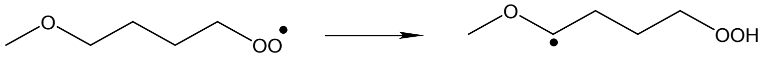 |
| [O]OCCOC(C)C → C[C](C)OCCOO | P-Ta' | 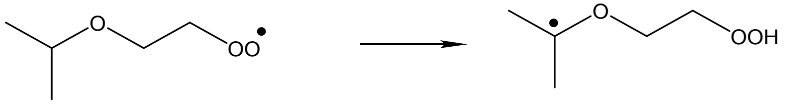 |
| [O]OCCCC(C)OC → CO[C](C)CCCOO | P-Ta | 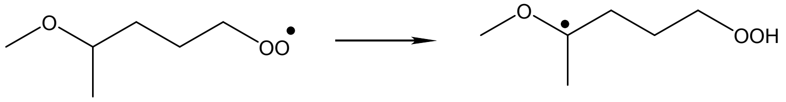 |
| [O]OCOCC(C)C → C[C](C)COCOO | Pa-T’ | 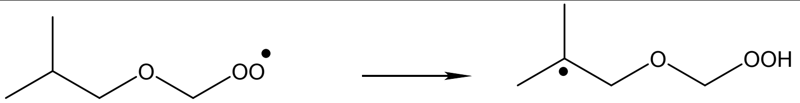 |
| [O]OC(C)OCC → [CH2]COC(C)OO | Sa-P' | 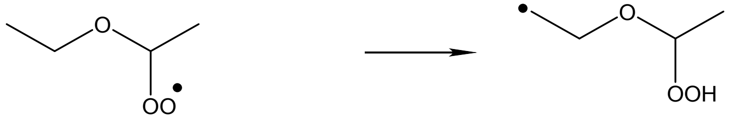 |

| | | |
|---|---|---|
| [O]OC(OC)CCC → [CH2]CCC(OC)OO | Sa-P | 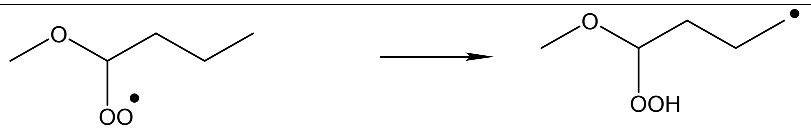 |
| [O]OC(C)COC → [CH2]OCC(C)OO | S-Pa’ | 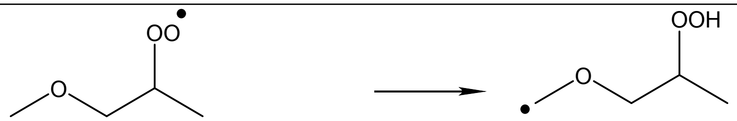 |
| [O]OC(C)COCC → C[CH]OCC(C)OO | S-Sa' | 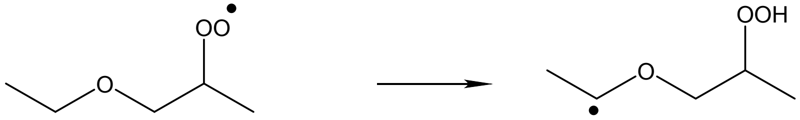 |
| [O]OC(C)CCCOC → CO[CH]CCC(C)OO | S-Sa | 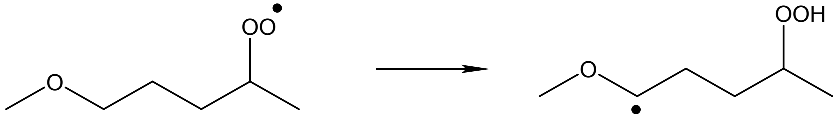 |
| [O]OC(C)OCCC → C[CH]COC(C)OO | Sa-S' | 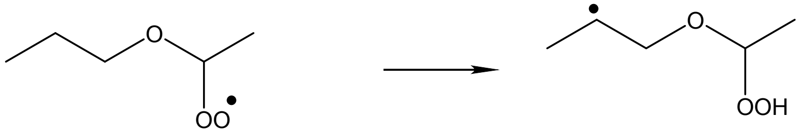 |
| [O]OC(OC)CCCC → C[CH]CCC(OC)OO | Sa-S | 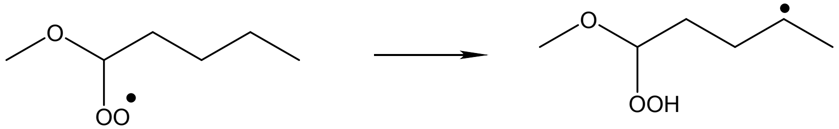 |
| [O]OC(C)COC(C)C → C[C](C)OCC(C)OO | S-Ta' | 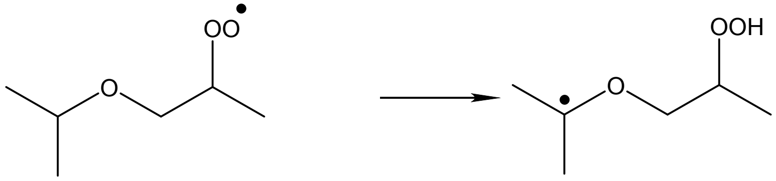 |

| | | |
|---|---|---|
| [O]OC(C)CCC(C)OC → CO[C](C)CCC(C)OO | S-Ta | 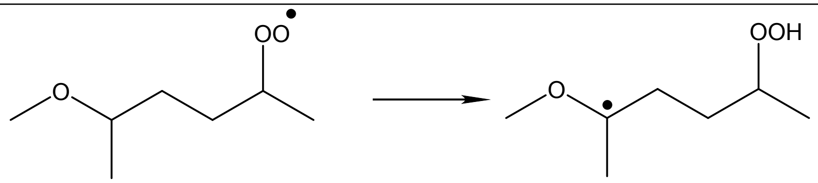 |
| [O]OC(C)OCC(C)C → C[C](C)COC(C)OO | Sa-T' | 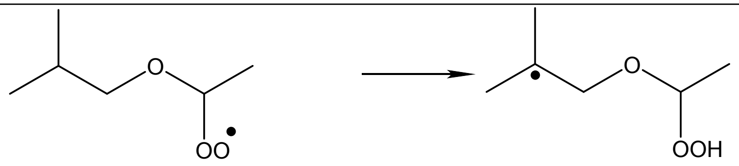 |
| [O]OC(OC)CCC(C)C → COC(CC[C](C)C)OO | Sa-T | 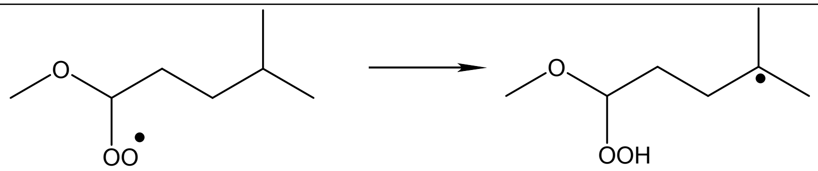 |
| [O]OC(C)(C)COC → [CH2]OCC(C)(C)OO | T-Pa' | 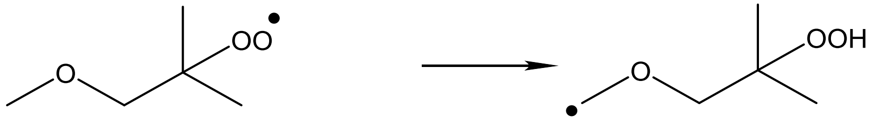 |
| [O]OC(C)(C)OCC → [CH2]COC(C)(C)OO | Ta-P' | 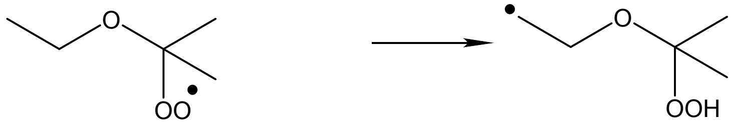 |
| [O]OC(OC)(C)CCC → [CH2]CCC(C)(OC)OO | Ta-P | 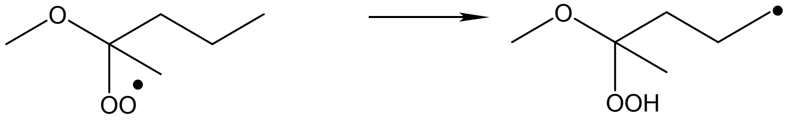 |
| [O]OC(C)(C)COCC → C[CH]OCC(C)(C)OO | T-Sa' | 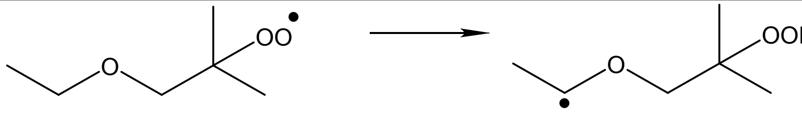 |

| | | |
|---|---|---|
| [O]OC(C)(C)CCCOC → CO[CH]CCC(C)(C)OO | T-Sa | 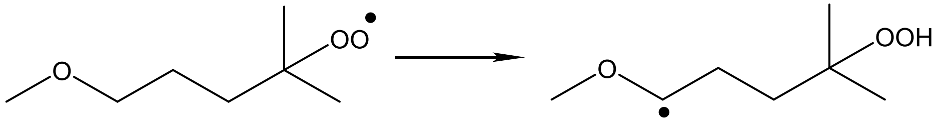 |
| [O]OC(C)(C)OCCC → C[CH]COC(C)(C)OO | Ta-S' | 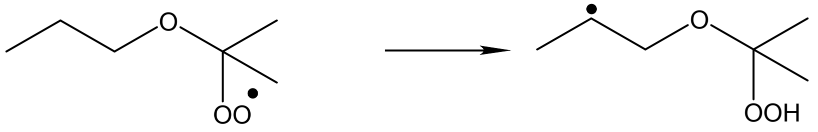 |
| [O]OC(OC)(C)CCCC → C[CH]CCC(C)(OC)OO | Ta-S | 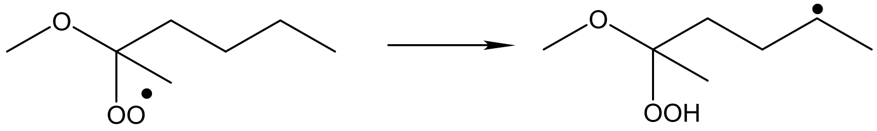 |
| [O]OC(C)(C)COC(C)C → C[C](C)OCC(C)(C)OO | T-Ta' | 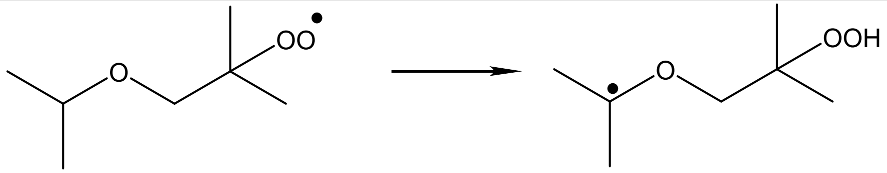 |
| [O]OC(C)(C)CCC(C)OC → CO[C](C)CCC(C)(C)OO | T-Ta | 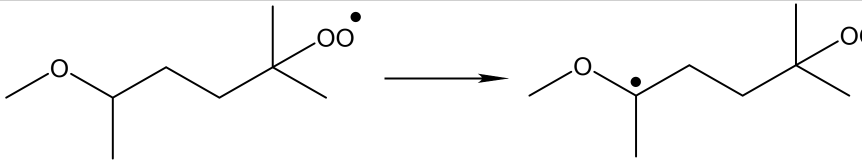 |
| [O]OC(C)(C)OCC(C)C → C[C](C)COC(C)(C)OO | Ta-T' | 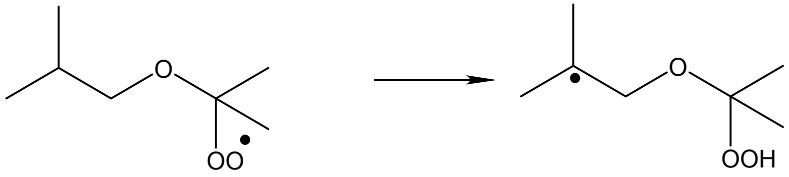 |
| [O]OC(OC)(C)CCC(C)C → COC(C)(CC[C](C)C)OO | Ta-T | 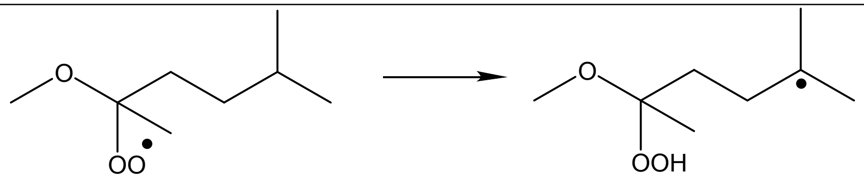 |
| **8-membered ring (N = 8)** | | |

| | | |
|---|---|---|
| [O]OCOCCC → [CH2]CCOCOO | $P_a$-P' | 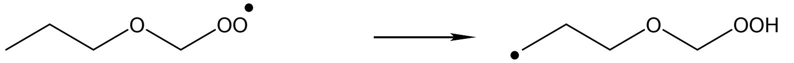 |
| [O]OCCCOC → [CH2]OCCCOO | P-$P_a$' | 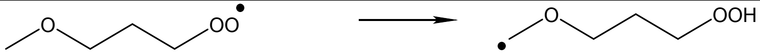 |
| [O]OCCCOCC → C[CH]OCCCOO | P-$S_a$' | 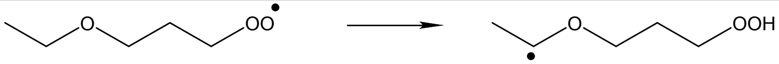 |
| [O]OCCCCCOC → CO[CH]CCCCOO | P-$S_a$ | 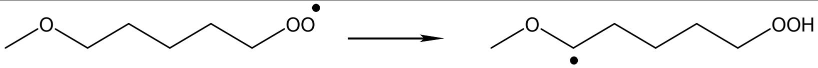 |
| [O]OCOCCCC → C[CH]CCOCOO | $P_a$-S' | 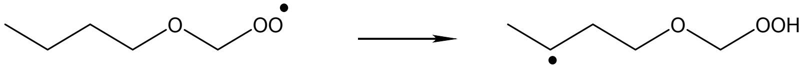 |
| [O]OCCCOC(C)C → C[C](C)OCCCOO | P-$T_a$' | 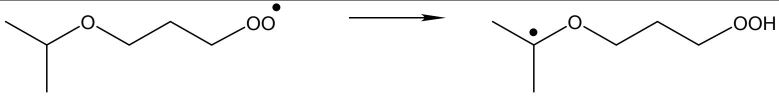 |
| [O]OCCCCC(C)OC → CO[C](C)CCCCOO | P-$T_a$ | 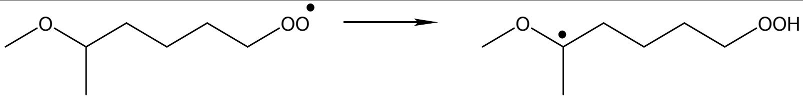 |
| [O]OCOCCC(C)C → C[C](C)CCOCOO | $P_a$-T' | 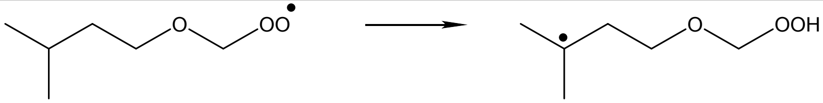 |
| [O]OC(C)OCCC → [CH2]CCOC(C)OO | $S_a$-P' | 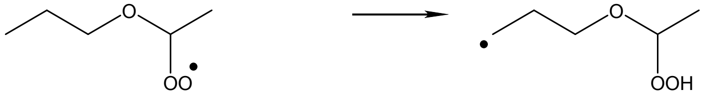 |
| [O]OC(CCCC)OC → [CH2]CCCC(OC)OO | $S_a$-P | 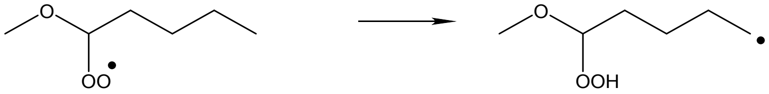 |
| [O]OC(C)CCOC → [CH2]OCCC(C)OO | S-$P_a$' | 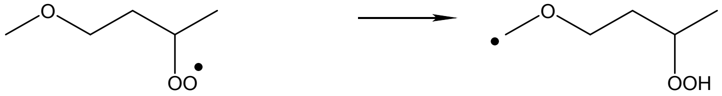 |

| | | |
|---|---|---|
| [O]OC(C)CCOCC → C[CH]OCCC(C)OO | S-$S_a$' | 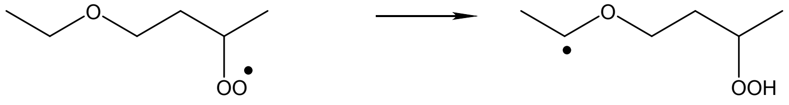 |
| [O]OC(C)CCCCOC → CO[CH]CCCC(C)OO | S-$S_a$ | 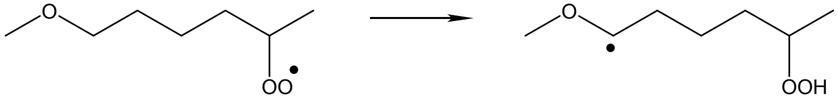 |
| [O]OC(C)OCCCC → C[CH]CCOC(C)OO | $S_a$-S' | 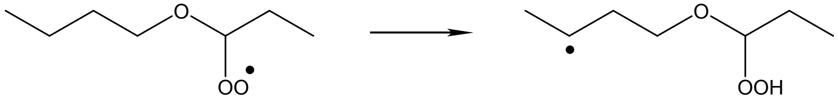 |
| [O]OC(CCCCC)OC → C[CH]CCCC(OC)OO | $S_a$-S | 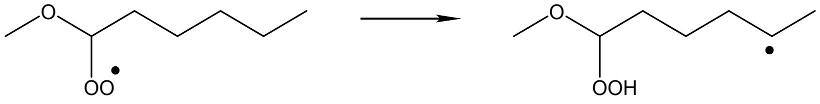 |
| [O]OC(C)CCOC(C)C → C[C](C)OCCC(C)OO | S-$T_a$' | 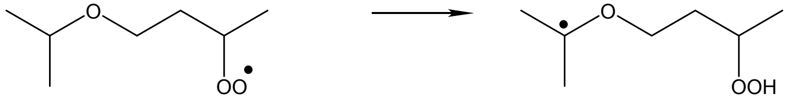 |
| [O]OC(C)CCCC(C)OC → CO[C](C)CCCC(C)OO | S-$T_a$ | 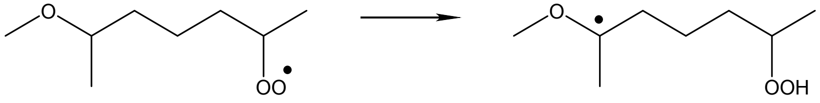 |
| [O]OC(C)OCCC(C)C → C[C](C)CCOC(C)OO | $S_a$-T' | 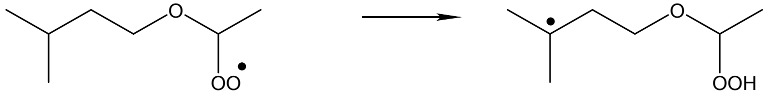 |
| [O]OC(CCCC(C)C)OC → COC(CCC[C](C)C)OO | $S_a$-T | 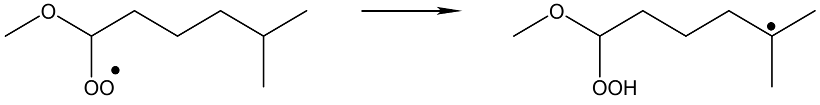 |
| [O]OC(C)(C)OCCC → [CH2]CCOC(C)(C)OO | $T_a$-P' | 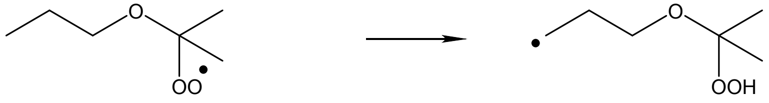 |
| [O]OC(C)(CCCC)OC → [CH2]CCCC(C)(OC)OO | $T_a$-P | 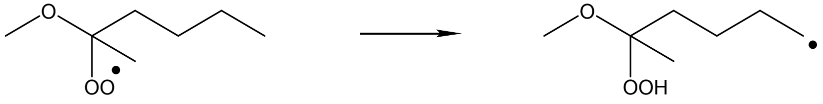 |

| | | |
|---|---|---|
| [O]OC(C)(C)CCOC → [CH2]OCCC(C)(C)OO | T-$P_a$' | 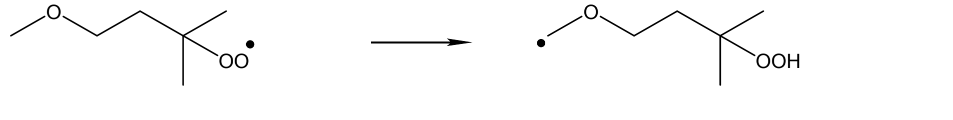 |
| [O]OC(C)(C)CCOCC → C[CH]OCCC(C)(C)OO | T-$S_a$' | 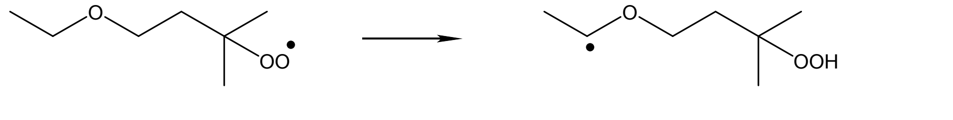 |
| [O]OC(C)(C)CCCCOC → CO[CH]CCCC(C)(C)OO | T-$S_a$ | 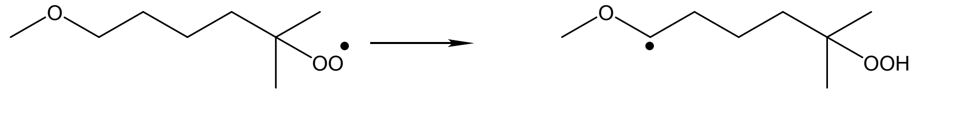 |
| [O]OC(C)(C)OCCCC → C[CH]CCOC(C)(C)OO | $T_a$-S' | 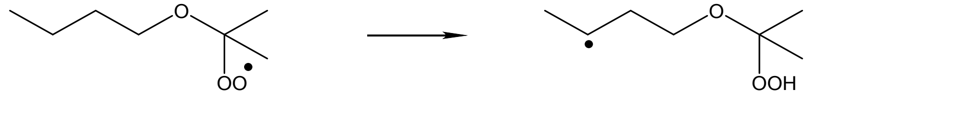 |
| [O]OC(C)(CCCCC)OC → C[CH]CCCC(C)(OC)OO | $T_a$-S | 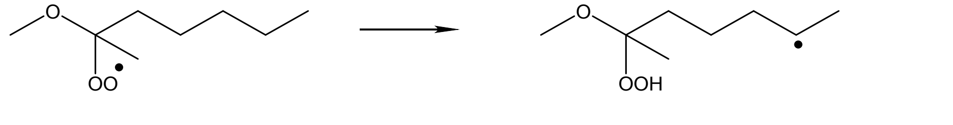 |
| [O]OC(C)(C)CCOC(C)C → C[C](C)OCCC(C)(C)OO | T-$T_a$' | 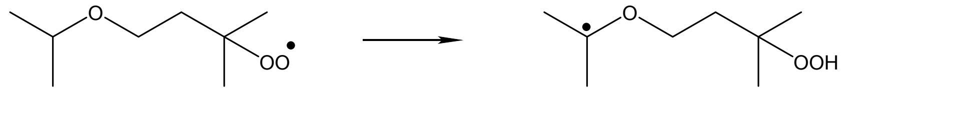 |
| [O]OC(C)(C)CCCC(C)OC → CO[C](C)CCCC(C)(C)OO | T-$T_a$ | 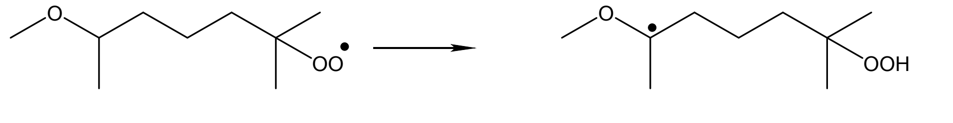 |
| [O]OC(C)(C)OCCC(C)C → C[C](C)CCOC(C)(C)OO | $T_a$-T' | 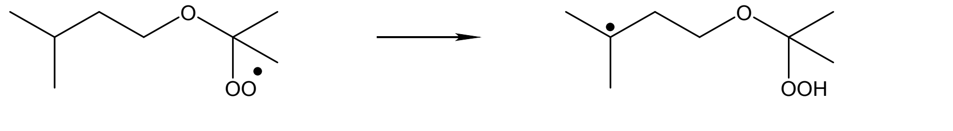 |
| [O]OC(C)(CCCC(C)C)OC → COC(C)(CCC[C](C)C)OO | $T_a$-T | 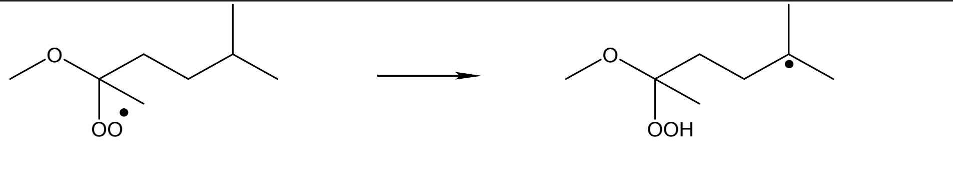 |
| [O]OCCOCC → [CH2]COCCOO | P-P' | 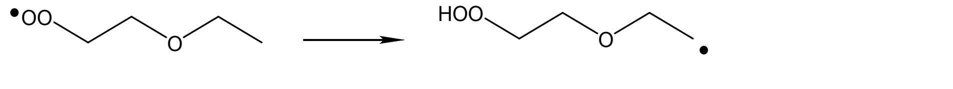 |

| | | |
|---|---|---|
| [O]OCCOCCC → C[CH]COCCOO | P-S' | 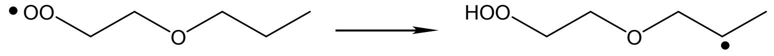 |
| [O]OCCOCC(C)C → C[C](C)COCCOO | P-T' | 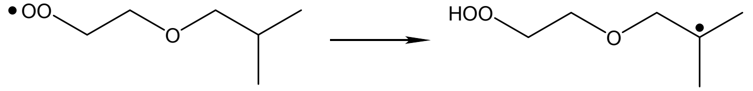 |
| [O]OC(C)COCC → [CH2]COCC(C)OO | S-P' | 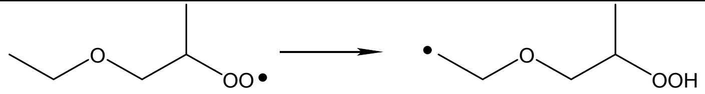 |
| [O]OC(C)COCCC → C[CH]COCC(C)OO | S-S' | 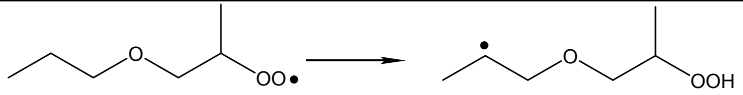 |
| [O]OC(C)COCC(C)C → C[C](C)COCC(C)OO | S-T' | 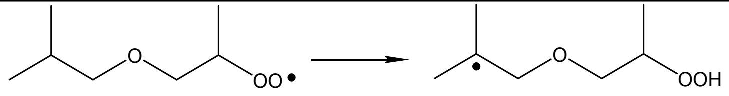 |
| [O]OC(C)(C)COCC → [CH2]COCC(C)(C)OO | T-P' | 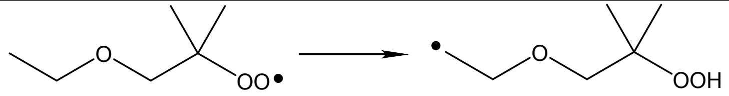 |
| [O]OC(C)(C)COCCC → C[CH]COCC(C)(C)OO | T-S' | 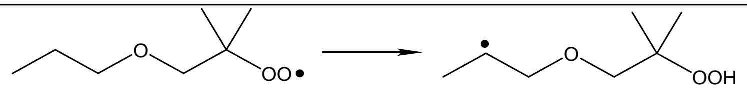 |
| [O]OC(C)(C)COCC(C)C → C[C](C)COCC(C)(C)OO | T-T' | 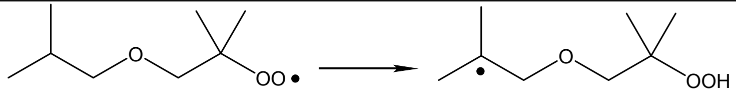 |
